# Existence and Uniqueness of the Local Smooth Solution to 3D Stochastic MHD Equations without Diffusion

**DOI:** 10.3390/e22010042

**Published:** 2019-12-27

**Authors:** Zhaoyang Qiu, Yanbin Tang

**Affiliations:** School of Mathematics and Statistics, Hubei Key Laboratory of Engineering Modeling and Scientific Computing, Huazhong University of Science and Technology, Wuhan 430074, China; zhqmath@gmail.com

**Keywords:** stochastic magneto-hydrodynamic equations, smooth solution, The Kato method, stopping time, progressive measurability

## Abstract

In this paper, we consider the existence of local smooth solution to stochastic magneto-hydrodynamic equations without diffusion forced by additive noise in $R^{3}$. We first transform the system into a random system via a simple change of variable and borrow the result obtained for classical magneto-hydrodynamic equations, then we show that this random transformed system is measurable with respect to the stochastic element. Finally we extend the solution to the maximality solution. Due to the coupled construction of this system, we need more elaborate and complicated estimates with respect to stochastic Euler equation.

## 1. Introduction

The magneto-hydrodynamic (MHD) equations have a wide range of applications in geophysics, astrophysics, and plasma physics [1,2,3,4,5,6,7,8,9,10]. Herein we consider the existence and uniqueness of the local smooth solution of the Cauchy problem to the following 3-dimensional (3D) stochastic MHD equations without diffusion,

(1) $$\begin{array}{c} \left\{\begin{array}{c} \frac{\partial u}{\partial t}+\left(u\cdot \nabla \right)u-\left(m\cdot \nabla \right)m+\nabla \left(\pi +\frac{{\left|m\right|}^{2}}{2}\right)=\sum_{j=1}^{\infty }g_{1,j}\left(t\right)\frac{d\beta_{j}^{1}\left(t\right)}{dt},\\ \frac{\partial m}{\partial t}+\left(u\cdot \nabla \right)m-\left(m\cdot \nabla \right)u=\sum_{j=1}^{\infty }g_{2,j}\left(t\right)\frac{d\beta_{j}^{2}\left(t\right)}{dt},\\ \nabla \cdot u=0,\;\nabla \cdot m=0,\\ u\left(0,x,\omega \right)=u_{0}\left(x,\omega \right),\;m\left(0,x,\omega \right)=m_{0}\left(x,\omega \right),x\in R^{3},\omega \in \Omega , \end{array}\right. \end{array}$$

where π, $u=(u_{1},u_{2},u_{3})$ and $m=(m_{1},m_{2},m_{3})$ denote, respectively, the pressure, velocity, and magnetic field. Assume that ${\left\{\beta_{j}^{1}\right\}}_{j=1}^{\infty }$ and ${\left\{\beta_{j}^{2}\right\}}_{j=1}^{\infty }$ are independent standard Brownian motions; they are defined in the filtered space $\left(\Omega ,F,{\left\{F_{t}\right\}}_{t\geq 0},P\right)$, which satisfy the natural assumption. The white noise driven terms in the system are natural for solving practical and theoretical problems. The symbol ω∈Ω for stochastic quantities will be understood throughout, but will be written explicitly hereafter.

If $g_{2,j}=0$ and m=0, Equation (1) will be reduced to the stochastic Euler equation. There are numerous references on the mathematical theory for stochastic Euler equation [11,12]. Bessaih [13] established the existence of martingale solution on bounded domain in $R^{2}$ by a compactness method. Brzezniak and Peszat [14] established the existence of martingale solution in $R^{2}$ by a viscosity vanishing method. Bessaih and Flandoli [15] studied 2D Euler equation perturbed by noise. Menaldi and Sritharan [16] considered 2D stochastic Navier–Stokes equation. Kim [17] discussed the 2D random Euler equations in a simply-connected bounded domain. Kim [18] established the existence of local smooth solution to 3D stochastic Euler equation forced by additive noise. Kim [19] considered the strong solutions of stochastic 3D Navier–Stokes equations. Glatt-Holtz and Vicol [20] established the local existence of smooth solution to 2D stochastic Euler equation driven by multiplicative noise with slip boundary conditions, and obtained the global existence of smooth solution forced by additive noise. The stochastic MHD equations were considered by many authors. Barbu and Da Prato [21] proved the existence of strong solution to 2D stochastic MHD equations in bounded domains, and the existence and uniqueness of an invariant measure were also obtained by the coupling method. The study on stochastic MHD equations, see also in [22,23,24,25] and references therein. Kim [26] established the existence and uniqueness of a local smooth solution to the stochastic initial value problem with $H^{\alpha }\left(R^{d}\right)-$initial data for $\alpha >\frac{d}{2}+2,d\geq 2$.

If $g_{1,j}=0$ and $g_{2,j}=0$, Equation (1) is reduced to the deterministic MHD equations without diffusion which is a special kind of quasi-linear symmetric hyperbolic system, as we known that it only has the local existence of smooth solution, see [27].

No one has addressed the existence of the local smooth solution to the stochastic MHD equations without diffusion driven by additive noise when the initial data belongs to $H^{\alpha }\left(R^{3}\right)$, $\alpha >\frac{5}{2}$. Therefore, we will extend the well-known result for the deterministic MHD equations to the corresponding stochastic case. For the stochastic case, the main difficulty comes from the nonlinear coupling terms as in deterministic case. To overcome this difficulty, we add a cut-off function depending on the size of ${∥\nabla \left(u,m\right)∥}_{L^{\infty }\left(R^{3}\right)}$ in front of the nonlinear convection term in the spirit of [18]. However, this cut-off function brings us an additional obstacle for uniqueness of the local smooth solution. Furthermore, we introduce a stopping time to overcome this new difficulty. Unlike the deterministic case, we need to show the measurability of solution obtained via a classical change of variable. To obtain suitable estimates, we need more elaborate and complicated estimates with respect to stochastic Euler equation due to the coupled construction of this system.

Our contributions are two-fold. First, we consider the stochastic MHD equations for initial data with spatial regularity actually only in $H^{\alpha }\left(R^{3}\right)$, $\alpha >\frac{5}{2}$, in contrast to Kim [26] with $H^{\alpha }\left(R^{d}\right)-$initial data for $\alpha >\frac{d}{2}+2,d\geq 2$. Then, the system (1) does not contain any diffusion terms.

We begin by reviewing some preliminaries associated with Equation (1) and then describe our main result. ∀s∈R, we define the usual Sobolev space

(2) $$\begin{array}{c} H^{s}\left(R^{3}\right)=\{f\in L^{2}\left(R^{3}\right)|(1+{\left|\xi \right|}^{2}{)}^{\frac{s}{2}}\hat{f}\left(\xi \right)\in L^{2}\left(R^{3}\right)\}, \end{array}$$

where $\hat{f}\left(\xi \right)$ is the Fourier transform of *f*, ${\langle \cdot ,\cdot \rangle }_{s}$ and ${∥\cdot ∥}_{s}$ are the inner product and the norm of $H^{s}\left(R^{3}\right)$, respectively. The Sobolev space $H_{\sigma }^{s}\left(R^{3}\right)$ is defined as follows,

(3) $$\begin{array}{c} H_{\sigma }^{s}\left(R^{3}\right)=\left\{f\in H^{s}\left(R^{3}\right)|\nabla \cdot f=0\right\}, \end{array}$$

which is a closed subspace of $H^{s}\left(R^{3}\right)$. The projection $\Pi :H^{s}\left(R^{3}\right)\to H_{\sigma }^{s}\left(R^{3}\right)$ can be expressed by means of the Fourier transform: for each $h=\left(h_{1},h_{2},h_{3}\right)\in H^{s}\left(R^{3}\right)$, we define ${\hat{\left(\Pi h\right)}}_{j}={\hat{h}}_{j}-\frac{\xi_{j}}{{\left|\xi \right|}^{2}}\sum_{k=1}^{3}\xi_{k}{\hat{h}}_{k}$, j=1,2,3. For $h=(h_{1},h_{2},h_{3})$, we write ${∥\nabla h∥}_{L^{\infty }\left(R^{3}\right)}=\sum_{i,j=1}^{3}{∥\partial_{i}h_{j}∥}_{L^{\infty }\left(R^{3}\right)}$ and

$${∥\nabla h∥}_{s}=\sum_{i,j=1}^{3}{∥\partial_{i}h_{j}∥}_{H^{s}\left(R^{3}\right)},\;\mathrm{if}\;\partial_{i}h_{j}=\frac{\partial h_{j}}{\partial x_{i}}\in H^{s}\left(R^{3}\right),\;\mathrm{for}\;\mathrm{all}\;\;i,j=1,2,3.$$

Define the trilinear form $\bar{B}:H_{\sigma }^{1}\times H_{\sigma }^{1}\times H_{\sigma }^{1}\to R$ by

$$\begin{array}{c} \bar{B}\left(Y_{1},Y_{2},Y_{3}\right)=b\left(u_{1},u_{2},u_{3}\right)-b\left(m_{1},m_{2},m_{3}\right)+b\left(u_{1},m_{2},m_{3}\right)-b\left(m_{1},u_{2},m_{3}\right), \end{array}$$

for all $Y_{i}=\left(u_{i},m_{i}\right)\in H_{\sigma }^{1}\times H_{\sigma }^{1},i=1,2,3$, where *b* is a continuous trilinear form given by

(4) $$\begin{array}{c} b\left(u,v,w\right)=\sum_{i,j=1}^{3}\int_{R^{3}}u_{i}\frac{\partial v_{j}}{\partial x_{i}}w_{j}dx \end{array}$$

satisfying the relations

(5) $$\begin{array}{c} b\left(u,v,v\right)=0,\;\mathrm{and}\;b\left(u,v,w\right)=-b\left(u,w,v\right),\forall \;u\in H_{\sigma }^{1},v,w\in H^{1}. \end{array}$$

Now we can define $B:H_{\sigma }^{1}\times H_{\sigma }^{1}\to {\left(H_{\sigma }^{1}\right)}^{*}$ as a continuous bilinear operator such that

$$\bar{B}\left(Y_{1},Y_{2},Y_{3}\right)=\langle B\left(Y_{1},Y_{2}\right),Y_{3}\rangle ,$$

where the ${\left(H_{\sigma }^{1}\right)}^{*}$ denotes the dual of $H_{\sigma }^{1}$. The existence of such an operator is guaranteed by the Riesz representation theorem. The notation 〈·,·〉 means the duality.

Denote the operators

$$\begin{array}{c} G_{j}=\left(\begin{array}{cc} g_{1,j} & 0\\ 0 & g_{2,j} \end{array}\right),W_{j}=\left(\begin{array}{c} \beta_{j}^{1}\\ \beta_{j}^{2} \end{array}\right), \end{array}$$

by the divergence free condition, system (1) can be rewritten as, in the sense of distributions in $H_{\sigma }^{1}$

(6) $$\begin{array}{c} \left\{\begin{array}{cc} dU+B\left(U\right)dt=\sum_{j=1}^{\infty }G_{j}\left(t\right)dW_{j}\left(t\right), & t>0,\\ U\left(0,x\right)=U_{0}\left(x\right), \end{array}\right. \end{array}$$

where $U={\left(u,m\right)}^{⊤}$, $U_{0}={\left(u_{0},m_{0}\right)}^{⊤}$, ⊤ denotes the transpose. Assume that ${\left\{G_{j}\right\}}_{j\geq 1}$ are $H^{\alpha }$-valued progressively measurable processes for some $\alpha >\frac{5}{2}$ such that for each T>0,

(7) $$\begin{array}{c} \sum_{j=1}^{\infty }E\int_{0}^{T}{∥G_{j}\left(t\right)∥}_{\alpha }^{2}dt<\infty . \end{array}$$

Now, we state the definition of a local smooth solution to the problem (1).

**Definition** **1.** *A pair (U,τ) is called a local smooth solution of (1) if the following conditions are satisfied.* *(1) U=U(t,ω) is an $H_{\sigma }^{\alpha }$-valued right continuous stochastic process adapted to ${\left\{F_{t}\right\}}_{t\geq 0}$, for some $\alpha >\frac{5}{2}$;* *(2) τ is a stopping time with respect to $\left\{F_{t}\right\}$, such that*

(8) $$\begin{array}{c} \tau \left(\omega \right)=\lim_{N\to \infty }\tau_{N}\left(\omega \right),\;\mathrm{for}\;\mathrm{almost}\;\mathrm{all}\;\omega , \end{array}$$

*where*

(9) $$\begin{array}{c} \tau_{N}\left(\omega \right)=\left\{\begin{array}{c} \inf\{0\leq t<\infty :∥\nabla U\left(t,\omega \right){∥}_{L^{\infty }\left(R^{3}\right)}\geq N\},\\ \infty ,\;if\;the\;above\;set\;\{\cdots \}\;is\;empty; \end{array}\right. \end{array}$$

*(3) $U\left(t,\omega \right)\in C(\left[0,\tau \left(\omega \right)\right);H_{\sigma }^{\alpha })$ satisfies for all 0≤t≤∞,N≥1*

(10) $$\begin{array}{c} U\left(t\wedge \tau_{N}\right)=U_{0}-\int_{0}^{t\wedge \tau_{N}}\Pi B\left(U\right)ds+\Pi \sum_{j=1}^{\infty }\int_{0}^{t\wedge \tau_{N}}G_{j}\left(s\right)dW_{j}\left(s\right),\;for\;almost\;all\;\omega . \end{array}$$

**Remark** **1.** *This is analogous to Definition 5.1 in [28]. By (8) and (9), we have τ=τ(ω)>0 for almost all ω.*

We now describe our main result.

**Theorem** **1.** *Let $U_{0}\in L^{2}\left(\Omega ;H_{\sigma }^{\alpha }\right)$ be $F_{0}$-measurable random variable and ${\left\{G_{j}\right\}}_{j\geq 1}$ satisfy (7). Thus, there exists a unique local smooth solution of (1) in the sense of Definition 1. Then, we obtain the estimate of τ:*

(11) $$\begin{array}{c} P\left\{\tau >\delta \right\}\geq 1-c\delta^{2}\left(E∥U_{0}{∥}_{\alpha }^{2}+\sum_{j=1}^{\infty }E\int_{0}^{\delta }{∥G_{j}\left(t\right)∥}_{\alpha }^{2}dt\right),\;\forall \;0<\delta <1, \end{array}$$

*where c>0 is a constant independent of U and δ. The solution is unique in the following sense; Suppose that $\left(U_{1},\tau \right)$ and $\left(U_{2},\tau \right)$ are local smooth solutions to (1), respectively, if $U_{0,1}\left(x\right)=U_{0,2}\left(x\right)=U_{0}\left(x\right)$ almost surely, then $P\left(U_{1}\left(t\wedge \tau \right)=U_{2}\left(t\wedge \tau \right);\forall t\geq 0\right)=1.$*

The paper is organized as follows. In Section 2, we construct the pathwise approximate smooth solution by introducing a cut-off function and controlling the nonlinear convection term in Equation (6) and regularizing the initial function and the noise with respect to the space variables. Equation (6) can be rewritten to a deterministic problem via a classical change of variable, then we apply the Kato method to get a smooth global solution for each fixed random element ω. To obtain the measurability of the solution, the continuous dependence on the initial data and the noise is established. In Section 3, by energy estimates and stopping time for fixed T>0 and N≥1 we first prove the existence of local smooth solution to the stochastic modified equations driven by an additive noise, then extend the existence interval by passing T→∞ and N→∞ where *N* ia a parameter in the cut-off function such that N=∞ makes the cut-off function become an identity map. The limit function will be the solution. Finally, by the Chebyshev inequality and energy estimates, the probability of existence can be made arbitrarily close to one.

## 2. Construction of Approximate Solution

Let $\rho_{\varepsilon }=\rho_{\varepsilon }\left(x\right),\varepsilon >0$ be the standard mollifier, and define

(12) $$\begin{array}{c} U_{0,\varepsilon }=U_{0}*\rho_{\varepsilon },\;\;Q_{\varepsilon }\left(t\right)=\Pi \sum_{j=1}^{\infty }\int_{0}^{t}G_{j}\left(s\right)*\rho_{\varepsilon }dW_{j}\left(s\right)=\left(q_{\varepsilon }^{1},q_{\varepsilon }^{2}\right), \end{array}$$

where the convolution be taken with respect to the space variable. Then, $Q_{\varepsilon }$ is an $H_{\sigma }^{\alpha }$-valued continuous square integral martingale for every α≥1. For each integer N≥1, we define $\Phi_{N}$ as follows,

$$\begin{array}{c} \Phi_{N}\left(\xi \right)=\left\{\begin{array}{cc} 1, & \mathrm{if}\;\xi <N,\\ 1+N-\xi , & \mathrm{if}\;N\leq \xi \leq N+1,\\ 0, & \mathrm{if}\;\xi >N+1. \end{array}\right. \end{array}$$

Fix N≥1,ε>0 and ω∈Ω, let $U_{0,\varepsilon }\in H_{\sigma }^{\alpha +1}$ and $Q_{\varepsilon }\in C\left(\left[0,\infty \right);H_{\sigma }^{\alpha +2}\right)$. Let $u=v+q_{\varepsilon }^{1},\;m=\tilde{m}+q_{\varepsilon }^{2}$ and $Y=(v,\tilde{m})$, then we define a nonlinear operator and a function as follows,

$$\begin{array}{c} A\left(t,Y\right)=\Phi_{N}(∥\nabla \left(Y+Q_{\varepsilon }\left(t\right)\right){∥}_{L^{\infty }\left(R^{3}\right)})\left(\begin{array}{cc} \Pi (v+q_{\varepsilon }^{1})\cdot \nabla & -\Pi (\tilde{m}+q_{\varepsilon }^{2})\cdot \nabla \\ -\Pi (\tilde{m}+q_{\varepsilon }^{2})\cdot \nabla & \Pi (v+q_{\varepsilon }^{1})\cdot \nabla \end{array}\right),\\ F\left(t,Y\right)=\Phi_{N}(∥\nabla \left(Y+Q_{\varepsilon }\left(t\right)\right){∥}_{L^{\infty }\left(R^{3}\right)})\left(\begin{array}{c} -\Pi \left(\left(v+q_{\varepsilon }^{1}\right)\cdot \nabla \right)q_{\varepsilon }^{1}+\Pi \left(\left(\tilde{m}+q_{\varepsilon }^{2}\right)\cdot \nabla \right)q_{\varepsilon }^{2}\\ -\Pi \left(\left(v+q_{\varepsilon }^{1}\right)\cdot \nabla \right)q_{\varepsilon }^{2}+\Pi \left(\left(\tilde{m}+q_{\varepsilon }^{2}\right)\cdot \nabla \right)q_{\varepsilon }^{1} \end{array}\right). \end{array}$$

Therefore, Equation (1) can be rewritten as an abstract Cauchy problem

(13) $$\begin{array}{c} \left\{\begin{array}{c} Y^{\prime }\left(t\right)+A\left(t,Y\right)Y=F\left(t,Y\right),\\ Y\left(0\right)=U_{0,\varepsilon }. \end{array}\right. \end{array}$$

Let $\Lambda ={\left(I-\Delta \right)}^{\frac{1}{2}}$ and Δ be the Laplacian operator in $R^{3}$. To apply the Kato method to (13), we need to verify the properties of Λ,A(t,Y),F(t,Y) appearing in Theorem 6 in [27].

(1) Λ is an isomorphism of $H_{\sigma }^{\alpha +1}$ into $H_{\sigma }^{\alpha }$.

(2) $\forall Y\in H_{\sigma }^{\alpha +1}$ with ${∥Y∥}_{\alpha +1}\leq b$, and each t∈[0,T], A(t,Y) is quasi-*m*-accretive in $H_{\sigma }^{\alpha }$, see [29].

(3) $\forall Y\in H_{\sigma }^{\alpha +1}$ and t≥0, there is a bounded linear operator B(t,Y) on $H_{\sigma }^{\alpha }$, such that

$$\begin{array}{c} \Lambda A\left(t,Y\right)\Lambda^{-1}=A\left(t,Y\right)+B\left(t,Y\right),\;{∥B\left(t,Y\right)∥}_{L(H_{\sigma }^{\alpha })}\leq \lambda_{1}\left(b,T\right),\\ {∥B\left(t,Y\right)-B\left(t,Z\right)∥}_{L(H_{\sigma }^{\alpha })}\leq \mu_{1}\left(b,T\right){∥Y-Z∥}_{\alpha +1}, \end{array}$$

for all $Y,Z\in H_{\sigma }^{\alpha +1}$ with ${∥Y∥}_{\alpha +1},{∥Z∥}_{\alpha +1}\leq b$ and all t∈[0,T], where ${∥\cdot ∥}_{L(H_{\sigma }^{\alpha })}$ denotes the operator norm of bounded linear operators on $H_{\sigma }^{\alpha }$.

(4) $\forall Y\in H_{\sigma }^{\alpha +1}$ and t≥0, A(t,Y) is a bounded linear operator from $H_{\sigma }^{\alpha }$ into $H_{\sigma }^{\alpha +1}$, such that

$$\begin{array}{c} {∥A\left(t,Y\right)-A\left(t,Z\right)∥}_{L(H_{\sigma }^{\alpha +1},H_{\sigma }^{\alpha })}\leq \mu_{2}\left(b,T\right){∥Y-Z∥}_{\alpha }, \end{array}$$

for all $Y,Z\in H_{\sigma }^{\alpha +1}$ with ${∥Y∥}_{\alpha +1},{∥Z∥}_{\alpha +1}\leq b$ and all t∈[0,T], where ${∥\cdot ∥}_{L(H_{\sigma }^{\alpha +1},H_{\sigma }^{\alpha })}$ denotes the operator norm of bounded linear operator from $H_{\sigma }^{\alpha +1}$ into $H_{\sigma }^{\alpha }$.

(5) $\forall Y\in H_{\sigma }^{\alpha +1}$ with ${∥Y∥}_{\alpha +1}\leq b$ and t∈[0,T], F(t,Y) is $H_{\sigma }^{\alpha +1}$-valued and ${∥F\left(t,Y\right)∥}_{\alpha +1}\leq \lambda_{2}\left(b,T\right)$ and the map t↦F(t,Y) is continuous from [0,T] into $H_{\sigma }^{\alpha }$. Also, for all $Y,Z\in H_{\sigma }^{\alpha +1}$ with ${∥Y∥}_{\alpha +1},{∥Z∥}_{\alpha +1}\leq b$,

$$\begin{array}{ccc} {∥F\left(t,Y\right)-F\left(t,Z\right)∥}_{\alpha } & \leq & \mu_{3}\left(b,T\right){∥Y-Z∥}_{\alpha },\\ {∥F\left(t,Y\right)-F\left(t,Z\right)∥}_{\alpha +1} & \leq & \mu_{4}\left(b,T\right){∥Y-Z∥}_{\alpha +1}. \end{array}$$

The above $\lambda_{i}=\lambda_{i}\left(b,T\right),i=1,2$ and $\mu_{i}=\mu_{i}\left(b,T\right),i=1,2,3,4$ are certain non-negative functions defined for b≥0 and T>0, which are nondecreasing in *r* and *T*.

Property (1) follows from the fact that Λ commutes with Π. Property (3) was established in [27] when $\Phi_{N}=1$ and $Q_{\varepsilon }=1$. Note that $\Phi_{N}$ is independent of space variable and $0\leq \Phi_{N}\leq 1$. At the same time, $Q_{\varepsilon }\in C\left(\left[0,\infty \right);H_{\sigma }^{\alpha +2}\right)$ is a given function and it plays the same role as *Y*. Therefore, the method in [30] can be applicable to property (3). Property (4) holds due to the following inequality,

$$\begin{array}{c} ∥\Phi_{N}(∥\nabla \left(Y_{1}+Q_{\varepsilon }\left(t\right)\right){∥}_{L^{\infty }\left(R^{3}\right)})\Pi \left(\left(v_{1}+q_{\varepsilon }^{1}\right)\cdot \right)\nabla )\\ \;-\Phi_{N}(∥\nabla \left(Y_{2}+Q_{\varepsilon }\left(t\right)\right){∥}_{L^{\infty }\left(R^{3}\right)})\Pi \left(\left(v_{2}+q_{\varepsilon }^{1}\right)\cdot \right)\nabla {)∥}_{L(H_{\sigma }^{\alpha +1},H_{\sigma }^{\alpha })}\\ +∥\Phi_{N}(∥\nabla \left(Y_{1}+Q_{\varepsilon }\left(t\right)\right){∥}_{L^{\infty }\left(R^{3}\right)})\Pi \left(\left({\tilde{m}}_{1}+q_{\varepsilon }^{2}\right)\cdot \right)\nabla )\\ \;-\Phi_{N}(∥\nabla \left(Y_{2}+Q_{\varepsilon }\left(t\right)\right){∥}_{L^{\infty }\left(R^{3}\right)})\Pi \left(\left({\tilde{m}}_{2}+q_{\varepsilon }^{2}\right)\cdot \right)\nabla {)∥}_{L(H_{\sigma }^{\alpha +1},H_{\sigma }^{\alpha })}\\ \leq C∥\nabla \left(Y_{1}-Y_{2}\right){∥}_{L^{\infty }\left(R^{3}\right)}(∥v_{1}{∥}_{\alpha }+∥q_{\varepsilon }^{1}{∥}_{\alpha })+C∥v_{1}-v_{2}{∥}_{\alpha }\\ \;+C∥\nabla \left(Y_{1}-Y_{2}\right){∥}_{L^{\infty }\left(R^{3}\right)}(∥{\tilde{m}}_{1}{∥}_{\alpha }+∥q_{\varepsilon }^{2}{∥}_{\alpha })+C∥{\tilde{m}}_{1}-{\tilde{m}}_{2}{∥}_{\alpha }\\ \leq {C(∥Y∥}_{\alpha }+∥Q_{\varepsilon }{∥}_{\alpha }+C)∥Y_{1}-Y_{2}{∥}_{\alpha }, \end{array}$$

for some positive constant *C* independent of $Y_{1},Y_{2},Q_{\varepsilon }$.

By the fact that $Q_{\varepsilon }\in C\left(\left[0,\infty \right);H_{\sigma }^{\alpha +2}\right)$ and similar estimates, we can obtain property (5).

With these properties in hand, we apply Theorem 6 in [27] to obtain a local existence to the Cauchy problem (13). In order to extend the local solution to a global solution in time, we will need the following lemmas.

**Lemma** **1.** *[31] Let w be a Lipschitz continuous function in $R^{3}$ and $v\in L^{2}\left(R^{3}\right)$. Then,*

$$\begin{array}{c} ∥\left(w\partial_{j}v\right)*\rho_{\varepsilon }-w\left(\partial_{j}v*\rho_{\varepsilon }\right){∥}_{L^{2}\left(R^{3}\right)}\leq {c∥\nabla w∥}_{L^{\infty }\left(R^{3}\right)}{∥v∥}_{L^{2}\left(R^{3}\right)}, \end{array}$$

*for some constant c independent of ε>0,v,w, and ∀v,w, the left-hand side tends to zero as ε→0.*

**Lemma** **2.** *[30] Let $f,g\in H^{s}\left(R^{3}\right)$, for some $s>\frac{5}{2}$. Then*

$$\begin{array}{c} ∥\Lambda^{s}\left(fg\right)-f\left(\Lambda^{s}g\right){∥}_{L^{2}\left(R^{3}\right)}\leq {c(∥\nabla f∥}_{L^{\infty }\left(R^{3}\right)}{∥g∥}_{H^{s-1}\left(R^{3}\right)}+{∥g∥}_{L^{\infty }\left(R^{3}\right)}{∥f∥}_{H^{s}\left(R^{3}\right)}), \end{array}$$

*for a constant c>0 independent of f,g.*

**Proposition** **1.** *If $Y_{0,\varepsilon }\in H_{\sigma }^{\alpha +1}$ and $Q_{\varepsilon }\in C\left(\left[0,\infty \right);H_{\sigma }^{\alpha +2}\right)$, then for any fixed N≥1, there is a unique solution $Y\in C(\left[0,\infty \right);H_{\sigma }^{\alpha +1})\cap$$C^{1}\left(\left[0,\infty \right);H_{\sigma }^{\alpha }\right)$ of (13) for ε>0 and ω∈Ω.*

**Proof.**  Firstly, using Lemma 1 and Lemma 2, $\forall u,v\in H_{\sigma }^{\alpha +1}$, we have the estimates

(14) $$\begin{array}{ccc} \; & \; & ∥\Pi \left(\Lambda^{\alpha +1}\left(u\cdot \nabla \right)v\right)*\rho_{\delta }-\Pi \left(\left(u\cdot \nabla \right)\Lambda^{\alpha +1}v\right)*\rho_{\delta }{∥}_{0}\\ \; & \; & \leq {c∥\nabla u∥}_{L^{\infty }\left(R^{3}\right)}{∥v∥}_{\alpha +1}+{c∥u∥}_{\alpha +1}{∥\nabla v∥}_{L^{\infty }\left(R^{3}\right)}, \end{array}$$

(15) $$\begin{array}{ccc} \; & \; & ∥\Pi \left(\left(u\cdot \nabla \right)\Lambda^{\alpha +1}v\right)*\rho_{\delta }-\Pi \left(\left(u\cdot \nabla \right)\left(\Lambda^{\alpha +1}v*\rho_{\delta }\right)\right){∥}_{0}\leq {c∥\nabla u∥}_{L^{\infty }\left(R^{3}\right)}{∥v∥}_{\alpha +1}, \end{array}$$

(16) $$\begin{array}{ccc} \; & \; & |{\langle \Pi \left(\left(u\cdot \nabla \right)\left(\Lambda^{\alpha +1}v*\rho_{\delta }\right)\right),\Lambda^{\alpha +1}v*\rho_{\delta }\rangle }_{0}{|\leq c∥\nabla u∥}_{L^{\infty }\left(R^{3}\right)}{∥v∥}_{\alpha +1}^{2}. \end{array}$$

Due to the work in [27], there is a local solution $Y\in C\left(\left[0,T\right];H_{\sigma }^{\alpha +1}\right)\cap C^{1}\left(\left[0,T\right];H_{\sigma }^{\alpha }\right)$ for some T>0. Therefore, ∀δ>0, the solution *Y* satisfies

(17) $$\begin{array}{ccc} \; & \; & \frac{\partial v*\rho_{\delta }}{\partial t}+\Phi_{N}(∥\nabla \left(Y+Q_{\varepsilon }\left(t\right)\right){∥}_{L^{\infty }\left(R^{3}\right)})\Pi \left(\left(\left(v+q_{\varepsilon }^{1}\right)\cdot \nabla \right)v-\left(\left(\tilde{m}+q_{\varepsilon }^{2}\right)\cdot \nabla \right)\tilde{m}\right)*\rho_{\delta }\\ \; & \; & +\Phi_{N}(∥\nabla \left(Y+Q_{\varepsilon }\left(t\right)\right){∥}_{L^{\infty }\left(R^{3}\right)})\Pi \left(\left(\left(v+q_{\varepsilon }^{1}\right)\cdot \nabla \right)q_{\varepsilon }^{1}-\left(\left(\tilde{m}+q_{\varepsilon }^{2}\right)\cdot \nabla \right)q_{\varepsilon }^{2}\right)*\rho_{\delta }=0, \end{array}$$

(18) $$\begin{array}{ccc} \; & \; & \frac{\partial \tilde{m}*\rho_{\delta }}{\partial t}+\Phi_{N}(∥\nabla \left(Y+Q_{\varepsilon }\left(t\right)\right){∥}_{L^{\infty }\left(R^{3}\right)})\Pi \left(\left(\left(v+q_{\varepsilon }^{1}\right)\cdot \nabla \right)\tilde{m}-\left(\left(\tilde{m}+q_{\varepsilon }^{2}\right)\cdot \nabla \right)v\right)*\rho_{\delta }\\ \; & \; & +\Phi_{N}(∥\nabla \left(Y+Q_{\varepsilon }\left(t\right)\right){∥}_{L^{\infty }\left(R^{3}\right)})\Pi \left(\left(\left(v+q_{\varepsilon }^{1}\right)\cdot \nabla \right)q_{\varepsilon }^{2}-\left(\left(\tilde{m}+q_{\varepsilon }^{2}\right)\cdot \nabla \right)q_{\varepsilon }^{1}\right)*\rho_{\delta }=0. \end{array}$$

By virtue of Equations (5) and (14)–(16), we have

(19) $$\begin{array}{c} \begin{array}{c} |{\langle \Pi \Lambda^{\alpha +1}\left(\left(\left(v+q_{\varepsilon }^{1}\right)\cdot \nabla \right)v-\left(\left(\tilde{m}+q_{\varepsilon }^{2}\right)\cdot \nabla \right)\tilde{m}\right)*\rho_{\delta },\Lambda^{\alpha +1}v*\rho_{\delta }\rangle }_{0}\\ \;+{\langle \Pi \Lambda^{\alpha +1}\left(\left(\left(v+q_{\varepsilon }^{1}\right)\cdot \nabla \right)\tilde{m}-\left(\left(\tilde{m}+q_{\varepsilon }^{2}\right)\cdot \nabla \right)v\right)*\rho_{\delta },\Lambda^{\alpha +1}\tilde{m}*\rho_{\delta }\rangle }_{0}|\\ \leq {C(∥\nabla v∥}_{L^{\infty }\left(R^{3}\right)}+∥\nabla q_{\varepsilon }^{1}{∥}_{L^{\infty }\left(R^{3}\right)}{)(∥v∥}_{\alpha +1}+∥q_{\varepsilon }^{1}{∥}_{\alpha +1}{)∥v∥}_{\alpha +1}\\ \;+C(∥\nabla \tilde{m}{∥}_{L^{\infty }\left(R^{3}\right)}+∥\nabla q_{\varepsilon }^{2}{∥}_{L^{\infty }\left(R^{3}\right)})(∥\tilde{m}{∥}_{\alpha +1}+∥q_{\varepsilon }^{2}{∥}_{\alpha +1}{)∥v∥}_{\alpha +1}\\ \;+{C(∥\nabla v∥}_{L^{\infty }\left(R^{3}\right)}+∥\nabla q_{\varepsilon }^{1}{∥}_{L^{\infty }\left(R^{3}\right)}+∥\nabla \tilde{m}{∥}_{L^{\infty }\left(R^{3}\right)}{)(∥v∥}_{\alpha +1}+∥q_{\varepsilon }^{1}{∥}_{\alpha +1}+∥\tilde{m}{∥}_{\alpha +1})∥\tilde{m}{∥}_{\alpha +1}\\ \;+C(∥\nabla \tilde{m}{∥}_{L^{\infty }\left(R^{3}\right)}+∥\nabla q_{\varepsilon }^{2}{∥}_{L^{\infty }\left(R^{3}\right)}+{∥\nabla v∥}_{L^{\infty }\left(R^{3}\right)})(∥\tilde{m}{∥}_{\alpha +1}+∥q_{\varepsilon }^{2}{∥}_{\alpha +1}+{∥v∥}_{\alpha +1})∥\tilde{m}{∥}_{\alpha +1}, \end{array} \end{array}$$

for some constant *C* independent of $v,\tilde{m},q_{\varepsilon }^{1},q_{\varepsilon }^{2}$, and δ.

On the other hand, we can estimate directly

(20) $$\begin{array}{ccc} \; & \; & |{\langle \Pi \Lambda^{\alpha +1}\left(\left(\left(v+q_{\varepsilon }^{1}\right)\cdot \nabla \right)q_{\varepsilon }^{1}-\left(\left(\tilde{m}+q_{\varepsilon }^{2}\right)\cdot \nabla \right)q_{\varepsilon }^{2}\right)*\rho_{\delta },\Lambda^{\alpha +1}v*\rho_{\delta }\rangle }_{0}|\\ \; & \leq & C(∥q_{\varepsilon }^{1}{∥}_{\alpha +2}+∥q_{\varepsilon }^{2}{∥}_{\alpha +2}{)(∥v∥}_{\alpha +1}+∥q_{\varepsilon }^{1}{∥}_{\alpha +1}+∥\tilde{m}{∥}_{\alpha +1}+∥q_{\varepsilon }^{2}{∥}_{\alpha +1}{)∥v∥}_{\alpha +1}, \end{array}$$

(21) $$\begin{array}{ccc} \; & \; & |{\langle \Pi \Lambda^{\alpha +1}\left(\left(\left(v+q_{\varepsilon }^{1}\right)\cdot \nabla \right)q_{\varepsilon }^{2}-\left(\left(\tilde{m}+q_{\varepsilon }^{2}\right)\cdot \nabla \right)q_{\varepsilon }^{1}\right)*\rho_{\delta },\Lambda^{\alpha +1}\tilde{m}*\rho_{\delta }\rangle }_{0}|\\ \; & \leq & C(∥q_{\varepsilon }^{1}{∥}_{\alpha +2}+∥q_{\varepsilon }^{2}{∥}_{\alpha +2}{)(∥v∥}_{\alpha +1}+∥q_{\varepsilon }^{1}{∥}_{\alpha +1}+∥\tilde{m}{∥}_{\alpha +1}+∥q_{\varepsilon }^{2}{∥}_{\alpha +1})∥\tilde{m}{∥}_{\alpha +1}, \end{array}$$

for some constants *C* independent of $v,\tilde{m},q_{\varepsilon }^{1},q_{\varepsilon }^{2}$, and δ. Applying $\Lambda^{\alpha +1}$ on (17), (18) and multiplying by $v*\rho_{\delta }$ and $\tilde{m}*\rho_{\delta }$, respectively; then, using (19)–(21) and passing δ→0, we obtain

(22) $$\begin{array}{ccc} {∥Y\left(t\right)∥}_{\alpha +1}^{2} & \leq & ∥Y_{0}{∥}_{\alpha +1}^{2}+C\left(1+\underset{0\leq s\leq t}{\sup}{∥Q_{\varepsilon }\left(s\right)∥}_{\alpha +2}\right)\left(\int_{0}^{t}{∥Y\left(s\right)∥}_{\alpha +1}^{2}ds+\int_{0}^{t}{∥Q_{\varepsilon }\left(s\right)∥}_{\alpha +1}^{2}ds\right), \end{array}$$

for all t∈[0,T] and some positive constant *C* independent of *T*. By the Grönwall inequality, (22) yields that ${∥Y\left(t\right)∥}_{\alpha +1}$ is bounded on each bounded time interval. Therefore, we can extend the solution *Y* to a global one. □

We prove the measurability of *Y* as a function of ω∈Ω.

**Proposition** **2.** *∀T>0, the map ω↦Y from Ω into $C(\left[0,T\right];H_{\sigma }^{\alpha +1})$ is $F_{T}$-measurable.*

**Proof.**  Because of the fact that the map $\omega \mapsto (U_{0,\varepsilon },Q_{\varepsilon })$ from Ω into $H_{\sigma }^{\alpha +1}\times C\left(\left[0,T\right];H_{\sigma }^{\alpha +2}\right)$ is $F_{T}$-measurable, we only need to show the continuous dependence of *Y* on $U_{0,\varepsilon }$ and $Q_{\varepsilon }$. Suppose that as n→∞,

(23) $$\begin{array}{c} U_{0}^{n}\to U_{0,\varepsilon }\;\;\mathrm{in}\;H_{\sigma }^{\alpha +1},\;\;Q^{n}\to Q_{\varepsilon }\;\;\mathrm{in}\;C\left(\left[0,T\right];H_{\sigma }^{\alpha +2}\right), \end{array}$$

for each T>0. Therefore, ${\left\{Q^{n}\right\}}_{n=1}^{\infty }$ is uniformly bounded in $C(\left[0,T\right];H_{\sigma }^{\alpha +2})$.

Define the nonlinear operators and functions as follows,

$$\begin{array}{ccc} A^{n}\left(t,Y\right) & = & \Phi_{N}(∥\nabla \left(Y+Q^{n}\left(t\right)\right){∥}_{L^{\infty }\left(R^{3}\right)})\left(\begin{array}{cc} \Pi (v+q^{1,n})\cdot \nabla & -\Pi (\tilde{m}+q^{2,n})\cdot \nabla \\ -\Pi (\tilde{m}+q^{2,n})\cdot \nabla & \Pi (v+q^{1,n})\cdot \nabla \end{array}\right),\\ F^{n}\left(t,Y\right) & = & \Phi_{N}(∥\nabla \left(Y+Q^{n}\left(t\right)\right){∥}_{L^{\infty }\left(R^{3}\right)})\left(\begin{array}{c} -\Pi \left(\left(v+q^{1,n}\right)\cdot \nabla \right)q^{1,n}+\Pi \left(\left(\tilde{m}+q^{2,n}\right)\cdot \nabla \right)q^{2,n}\\ -\Pi \left(\left(v+q^{1,n}\right)\cdot \nabla \right)q^{2,n}+\Pi \left(\left(\tilde{m}+q^{2,n}\right)\cdot \nabla \right)q^{1,n} \end{array}\right). \end{array}$$

Denote $B^{n}$ such that

(24) $$\begin{array}{c} \Lambda A^{n}\left(t,Y\right)\Lambda^{-1}=A^{n}\left(t,Y\right)+B^{n}\left(t,Y\right). \end{array}$$

Then, for each $Y\in H_{\sigma }^{\alpha +1}$ and t≥0 we have

$$\begin{array}{ccc} F^{n}\left(t,Y\right) & \to & F\left(t,Y\right)\;\;\mathrm{in}\;H_{\sigma }^{\alpha +1},\\ A^{n}\left(t,Y\right) & \to & A\left(t,Y\right)\;\;\mathrm{strongly}\;\mathrm{in}\;L\left(H_{\sigma }^{\alpha +1},H_{\sigma }^{\alpha }\right),\\ B^{n}\left(t,Y\right) & \to & B\left(t,Y\right)\;\;\mathrm{strongly}\;\mathrm{in}\;L\left(H_{\sigma }^{\alpha },H_{\sigma }^{\alpha }\right), \end{array}$$

uniformly in *n*.

Let $Y^{n}$ be the solution of the problem

(25) $$\begin{array}{c} \left\{\begin{array}{c} \frac{dY^{n}}{dt}+A^{n}\left(t,Y^{n}\right)Y^{n}=F^{n}\left(t,Y^{n}\right),\\ Y^{n}\left(0\right)=U_{0}^{n}. \end{array}\right. \end{array}$$

Due to Theorem 7 of [27], there is some T>0 such that $Y^{n}\to Y$ in $C(\left[0,T\right];H_{\sigma }^{\alpha +1})$. ∀T>0, ${\left\{Y^{n}\right\}}_{n=1}^{\infty }$ is uniformly bounded in $C(\left[0,T\right];H_{\sigma }^{\alpha +1})$ by Equation (23). We can partition [0,T] into a number of smaller subintervals to get the continuous dependence of *Y* on $U_{0,\varepsilon }$ and $Q_{\varepsilon }$ on [0,T]. □

## 3. Existence and Uniqueness of the Local Smooth Solution

We now construct the pathwise smooth solution by means of approximate solutions obtained in Section 2.

**Step 1.** Construct the local smooth solution for any fixed N≥1 and T>0. 

Recalling (12) and (13), we choose a sequence $\left\{\varepsilon_{l}\right\}$ of decreasing positive numbers such that

(26) $$\begin{array}{c} U_{0,\varepsilon_{l}}\to U_{0}\;\;\mathrm{in}\;H_{\sigma }^{\alpha },\;\;Q_{\varepsilon_{l}}\to Q\;\;\mathrm{in}\;C\left(\left[0,T\right];H_{\sigma }^{\alpha }\right) \end{array}$$

as $\varepsilon_{l}\to 0$ for almost all ω, where

$$\begin{array}{c} Q\left(t\right)=\Pi \sum_{j=1}^{\infty }\int_{0}^{t}G_{j}\left(s\right)dW_{j}\left(s\right). \end{array}$$

Set $U_{l}=Y_{l}+Q_{\varepsilon_{l}}$, l=1,2,⋯, where $Y_{l}$ is a solution to (13) with $\varepsilon =\varepsilon_{l}$. Then, we have $U_{l}\in C\left(\left[0,T\right];H_{\sigma }^{\alpha +1}\right)$, for almost all ω and it is $H_{\sigma }^{\alpha +1}$-valued progressively measurable. It holds that

(27) $$\begin{array}{c} \frac{\partial U_{l}}{\partial t}+\Phi_{N}(∥\nabla U_{l}{∥}_{L^{\infty }\left(R^{3}\right)})\Pi B\left(U_{l}\right)=\frac{\partial Q_{\varepsilon_{l}}}{\partial t} \end{array}$$

in the sense of distributions over $R^{3}\times \left[0,\infty \right)$, for almost all ω.

We next define a stopping time $T_{l,K}$ for l,K=1,2,⋯ by

$$\begin{array}{c} T_{l,K}=\left\{\begin{array}{c} \inf\left\{0\leq t\leq T:∥U_{l}{∥}_{\alpha }\geq K\right\},\\ T,\;\mathrm{if}\;\mathrm{the}\;\mathrm{above}\;\mathrm{set}\;\{\cdots \}\;\mathrm{is}\;\mathrm{empty}. \end{array}\right. \end{array}$$

The Itô formula implies that

(28) $$\begin{array}{ccc} \; & \; & ∥u_{l}\left(t\wedge T_{l,K}\right){∥}_{\alpha }^{2}={∥u_{l}\left(0\right)∥}_{\alpha }^{2}\\ \; & \; & -2\int_{0}^{t\wedge T_{l,K}}\Phi_{N}(∥\nabla U_{l}{∥}_{L^{\infty }\left(R^{3}\right)}){\langle \left(u_{l}\left(s\right)\cdot \nabla \right)u_{l}\left(s\right)-\left(m_{l}\left(s\right)\cdot \nabla \right)m_{l}\left(s\right),u_{l}\left(s\right)\rangle }_{\alpha }ds\\ \; & \; & +2\sum_{j=1}^{\infty }\int_{0}^{t\wedge T_{l,K}}{\langle g_{1,j}\left(s\right)*\rho_{\varepsilon_{l}},u_{l}\left(s\right)\rangle }_{\alpha }d\beta_{j}^{1}\left(s\right)+\sum_{j=1}^{\infty }\int_{0}^{t\wedge T_{l,K}}{∥\Pi g_{1,j}\left(s\right)*\rho_{\varepsilon_{l}}∥}_{\alpha }^{2}ds,\; \end{array}$$

(29) $$\begin{array}{ccc} \; & \; & ∥m_{l}\left(t\wedge T_{l,K}\right){∥}_{\alpha }^{2}={∥m_{l}\left(0\right)∥}_{\alpha }^{2}\\ \; & \; & -2\int_{0}^{t\wedge T_{l,K}}\Phi_{N}(∥\nabla U_{l}{∥}_{L^{\infty }\left(R^{3}\right)}){\langle \left(u_{l}\left(s\right)\cdot \nabla \right)m_{l}\left(s\right)-\left(m_{l}\left(s\right)\cdot \nabla \right)u_{l}\left(s\right),m_{l}\left(s\right)\rangle }_{\alpha }ds\\ \; & \; & +2\sum_{j=1}^{\infty }\int_{0}^{t\wedge T_{l,K}}{\langle g_{2,j}\left(s\right)*\rho_{\varepsilon_{l}},m_{l}\left(s\right)\rangle }_{\alpha }d\beta_{j}^{2}\left(s\right)+\sum_{j=1}^{\infty }\int_{0}^{t\wedge T_{l,K}}{∥\Pi g_{2,j}\left(s\right)*\rho_{\varepsilon_{l}}∥}_{\alpha }^{2}ds,\; \end{array}$$

for all t≥0 and almost all ω. Using Equations (5) and (14), we can estimate the coupling terms

(30) $$\begin{array}{ccc} \; & \; & |\Phi_{N}(∥\nabla U_{l}{∥}_{L^{\infty }\left(R^{3}\right)}){\langle \left(u_{l}\left(s\right)\cdot \nabla \right)u_{l}\left(s\right)-\left(m_{l}\left(s\right)\cdot \nabla \right)m_{l}\left(s\right),u_{l}\left(s\right)\rangle }_{\alpha }\\ \; & \; & +\Phi_{N}(∥\nabla U_{l}{∥}_{L^{\infty }\left(R^{3}\right)}){\langle \left(u_{l}\left(s\right)\cdot \nabla \right)m_{l}\left(s\right)-\left(m_{l}\left(s\right)\cdot \nabla \right)u_{l}\left(s\right),m_{l}\left(s\right)\rangle }_{\alpha }|\\ \; & \; & \leq C_{N}(∥u_{l}{\left(s\right)∥}_{\alpha }^{2}+∥m_{l}\left(s\right){∥}_{\alpha }^{2}), \end{array}$$

for some constant $C_{N}>0$ independent of *l*. The Burkholder–Davis–Gundy inequality implies that

(31) $$\begin{array}{ccc} \; & \; & E\left(\underset{0\leq \xi \leq t}{\sup}|\sum_{j=1}^{\infty }\int_{0}^{\xi \wedge T_{l,K}}{\langle g_{1,j}\left(s\right)*\rho_{\varepsilon_{l}},u_{l}\left(s\right)\rangle }_{\alpha }d\beta_{j}^{1}\left(s\right)|\right)\\ \; & \; & +E\left(\underset{0\leq \xi \leq t}{\sup}|\sum_{j=1}^{\infty }\int_{0}^{\xi \wedge T_{l,K}}{\langle g_{2,j}\left(s\right)*\rho_{\varepsilon_{l}},m_{l}\left(s\right)\rangle }_{\alpha }d\beta_{j}^{2}\left(s\right)|\right)\\ \; & \leq & CE{\left(\sum_{j=1}^{\infty }\int_{0}^{t\wedge T_{l,K}}{\left|{\langle g_{1,j}\left(s\right)*\rho_{\varepsilon_{l}},u_{l}\left(s\right)\rangle }_{\alpha }\right|}^{2}ds\right)}^{\frac{1}{2}}\\ \; & \; & +CE{\left(\sum_{j=1}^{\infty }\int_{0}^{t\wedge T_{l,K}}{\left|{\langle g_{2,j}\left(s\right)*\rho_{\varepsilon_{l}},m_{l}\left(s\right)\rangle }_{\alpha }\right|}^{2}ds\right)}^{\frac{1}{2}}\\ \; & \leq & CE\left(\underset{0\leq s\leq t}{\sup}∥U_{l}\left(s\wedge T_{l,K}\right){∥}_{\alpha }(\sum_{j=1}^{\infty }\int_{0}^{t\wedge T_{l,K}}∥G_{j}\left(s\right)*\rho_{\varepsilon_{l}}{{∥}_{\alpha }^{2}ds)}^{\frac{1}{2}}\right)\\ \; & \leq & \frac{1}{2}E(\underset{0\leq s\leq t}{\sup}∥U_{l}\left(s\wedge T_{l,K}\right){∥}_{\alpha }^{2})+CE\left(\sum_{j=1}^{\infty }\int_{0}^{t\wedge T_{l,K}}{∥G_{j}\left(s\right)*\rho_{\varepsilon_{l}}∥}_{\alpha }^{2}ds\right), \end{array}$$

where C>0 is a constant independent of l,K and *t*. Combining (28)–(31), we have

(32) $$\begin{array}{c} E\left(\underset{0\leq s\leq T}{\sup}{∥U_{l}\left(s\wedge T_{l,K}\right)∥}_{\alpha }^{2}\right)\leq C_{N,T}, \end{array}$$

where the constant $C_{N,T}$ is independent of *l* and *K*. By the Fatou Lemma and passing K→∞, we get

(33) $$\begin{array}{c} E\left(\underset{0\leq t\leq T}{\sup}{∥U_{l}\left(t\right)∥}_{\alpha }^{2}\right)\leq C_{N,T}, \end{array}$$

thus we have $P\left\{\lim_{\bar{l\to \infty }}{∥U_{l}∥}_{C(\left[0,T\right];H_{\sigma }^{\alpha })}<\infty \right\}=1$, i.e., $\forall \;\omega \in \left\{\lim_{\bar{l\to \infty }}{∥U_{l}∥}_{C(\left[0,T\right];H_{\sigma }^{\alpha })}<\infty \right\}$, there exists L>0 such that $\omega \in \left\{\lim_{\bar{l\to \infty }}{∥U_{l}∥}_{C(\left[0,T\right];H_{\sigma }^{\alpha })}<L\right\}.$ Therefore, we consider the following set

$$\begin{array}{c} \Omega_{1}=\overset{\infty }{\underset{L=1}{⋃}}\overset{\infty }{\underset{k=1}{⋂}}\overset{\infty }{\underset{l=k}{⋃}}\left(∥U_{l}{∥}_{C(\left[0,T\right];H_{\sigma }^{\alpha })}\leq L\right). \end{array}$$

Let $\Omega_{2}$ be the set of all ω for which, as l→∞,

$$\begin{array}{c} U_{0,\varepsilon_{l}}\to U_{0}\;\;\mathrm{in}\;H_{\sigma }^{\alpha },\;\;Q_{\varepsilon_{l}}\to Q\;\;\mathrm{in}\;C\left(\left[0,T\right];H_{\sigma }^{\alpha }\right) \end{array}$$

and (27) holds in the sense of distributions over $R^{3}\times \left[0,\infty \right)$. Next, we define $\Omega^{*}=\Omega_{1}\cap \Omega_{2}$. Then, $P(\Omega^{*})=1$.

Now, fixed any $\omega^{*}\in \Omega^{*}$. Then, there exists some $L=L_{\omega^{*}}\geq 1$ and a subsequence denoted by $\left\{U_{l_{j}}\right\}$ such that

(34) $$\begin{array}{c} ∥U_{l_{j}}{∥}_{C(\left[0,T\right];H_{\sigma }^{\alpha })}\leq L, \end{array}$$

for all $l_{j}$. The choice of such a subsequence may depend on $\omega^{*}$.

As $U_{l_{j}}$ satisfies

(35) $$\begin{array}{c} \frac{\partial }{\partial t}\left(U_{l_{j}}-Q_{\varepsilon_{l_{j}}}\right)+\Phi_{N}(∥\nabla U_{l_{j}}{∥}_{L^{\infty }\left(R^{3}\right)})\Pi B\left(U_{l_{j}}\right)=0 \end{array}$$

in the sense of distributions over $R^{3}\times \left(0,\infty \right)$, thus

(36) $$\begin{array}{c} ∥\frac{\partial }{\partial t}\left(U_{l_{j}}-Q_{\varepsilon_{l_{j}}}\right){∥}_{C(\left[0,T\right];H_{\sigma }^{\alpha -1})}\leq C{∥U_{l_{j}}∥}_{C(\left[0,T\right];H_{\sigma }^{\alpha })}, \end{array}$$

where C>0 is a constant independent of $U_{l_{j}}$ and $\omega^{*}$. We choose a constant β>0 such that $\frac{5}{2}\vee \left(\alpha -1\right)<\beta <\alpha$. Combining (34) and (36), we extract a subsequence still denoted by $\left\{U_{l_{j}}\right\}$ such that

(37) $$\begin{array}{ccc} U_{l_{j}} & \to & U\;\;\mathrm{weak}\;\mathrm{star}\;\mathrm{in}\;L^{\infty }\left(0,T;H_{\sigma }^{\alpha }\right), \end{array}$$

(38) $$\begin{array}{ccc} U_{l_{j}} & \to & U\;\;\mathrm{strongly}\;\mathrm{in}\;C(\left[0,T\right];H^{\beta }\left(G\right)), \end{array}$$

for every bounded open ball *G* in $R^{3}$, as $l_{j}\to \infty$. Equation (38) uses the Corollary 8 in [32].

Next, we will prove that

(39) $$\begin{array}{c} \nabla U_{l_{j}}\to \nabla U\;\;\mathrm{strongly}\;\mathrm{in}\;C\left(\left[0,T\right];H^{\beta -1}\left(R^{3}\right)\right). \end{array}$$

It is enough to prove that

(40) $$\begin{array}{c} \nabla \left(\theta_{R}U_{l_{j}}\right)\to 0\;\;\mathrm{in}\;H^{\beta -1}\left(R^{3}\right), \end{array}$$

where $\theta \in C^{\infty }\left(R^{3}\right)$ such that θ(x)=1 for |x|≥2 and θ(x)=0 for |x|≤1. We define $\theta_{R}\left(x\right)=\theta \left(\frac{x}{R}\right)$, for R≥1. Then, (39) follows from (38) and (40).

**Lemma** **3.** *[18] Let s>0. For all $h\in H^{s}\left(R^{3}\right)$, it holds that*

(41) $$\begin{array}{c} ∥\theta_{R}{h∥}_{s}\leq {C∥h∥}_{s},\;\;∥\left(\nabla \theta_{R}\right){h∥}_{s}\leq \frac{C}{R}{∥h∥}_{s}, \end{array}$$

*for some constants C>0 independent of h and R≥1.*

As the interaction of $\theta_{R}$ with the projection operator Π is difficult to handle for our purpose, we remove Π by introducing the vorticity. Let ${\tilde{u}}_{l_{j}}=\nabla \times u_{l_{j}}$ and ${\tilde{m}}_{l_{j}}=\nabla \times m_{l_{j}}$. By Equation (27) and the equalities

(42) $$\begin{array}{ccc} \nabla \times [(u\cdot \nabla )u] & = & (u\cdot \nabla )(\nabla \times u)-((\nabla \times u)\cdot \nabla )u, \end{array}$$

(43) $$\begin{array}{ccc} \nabla \times [(u\cdot \nabla )m-(m\cdot \nabla )u] & = & (u\cdot \nabla )(\nabla \times m)-((\nabla \times m)\cdot \nabla )u\\ \; & - & (m\cdot \nabla )(\nabla \times u)+((\nabla \times u)\cdot \nabla )m-2f, \end{array}$$

where the vector function

(44) $$\begin{array}{c} f={\left(\frac{\partial m}{\partial x_{2}}\cdot \frac{\partial u}{\partial x_{3}}-\frac{\partial m}{\partial x_{3}}\cdot \frac{\partial u}{\partial x_{2}},\;\frac{\partial m}{\partial x_{3}}\cdot \frac{\partial u}{\partial x_{1}}-\frac{\partial m}{\partial x_{1}}\cdot \frac{\partial u}{\partial x_{3}},\;\frac{\partial m}{\partial x_{1}}\cdot \frac{\partial u}{\partial x_{2}}-\frac{\partial m}{\partial x_{2}}\cdot \frac{\partial u}{\partial x_{1}}\right)}^{T}, \end{array}$$

we have

(45) $$\begin{array}{ccc} \; & \; & \frac{\partial }{\partial t}\left(\theta_{R}{\tilde{u}}_{l_{j}}-\theta_{R}\nabla \times {\hat{q}}_{l_{j}}\right)+\Phi_{N}(∥\nabla U_{l_{j}}{∥}_{L^{\infty }\left(R^{3}\right)})\left(\left(u_{l_{j}}\cdot \nabla \right)\theta_{R}{\tilde{u}}_{l_{j}}-\left(m_{l_{j}}\cdot \nabla \right)\theta_{R}{\tilde{m}}_{l_{j}}\right)\\ \; & = & \Phi_{N}(∥\nabla U_{l_{j}}{∥}_{L^{\infty }\left(R^{3}\right)})(\left(\theta_{R}{\tilde{u}}_{l_{j}}\cdot \nabla \right)u_{l_{j}}-\left(\theta_{R}{\tilde{m}}_{l_{j}}\cdot \nabla \right)m_{l_{j}}\\ \; & \; & +\left(u_{l_{j}}\cdot \nabla \theta_{R}\right){\tilde{u}}_{l_{j}}-\left(m_{l_{j}}\cdot \nabla \theta_{R}\right){\tilde{m}}_{l_{j}}), \end{array}$$

(46) $$\begin{array}{ccc} \; & \; & \frac{\partial }{\partial t}\left(\theta_{R}{\tilde{m}}_{l_{j}}-\theta_{R}\nabla \times q_{l_{j}}\right)+\Phi_{N}(∥\nabla U_{l_{j}}{∥}_{L^{\infty }\left(R^{3}\right)})\left(\left(u_{l_{j}}\cdot \nabla \right)\theta_{R}{\tilde{m}}_{l_{j}}-\left(m_{l_{j}}\cdot \nabla \right)\theta_{R}{\tilde{u}}_{l_{j}}\right)\\ \; & = & \Phi_{N}(∥\nabla U_{l_{j}}{∥}_{L^{\infty }\left(R^{3}\right)})(\left(\theta_{R}{\tilde{u}}_{l_{j}}\cdot \nabla \right)m_{l_{j}}-\left(\theta_{R}{\tilde{m}}_{l_{j}}\cdot \nabla \right)u_{l_{j}}\\ \; & \; & +\left(u_{l_{j}}\cdot \nabla \theta_{R}\right){\tilde{m}}_{l_{j}}-\left(m_{l_{j}}\cdot \nabla \theta_{R}\right){\tilde{u}}_{l_{j}})+2\Phi_{N}(∥\nabla U_{l_{j}}{∥}_{L^{\infty }\left(R^{3}\right)})\theta_{R}f. \end{array}$$

Combining Equations (4), (5), (34); Lemma 3; and the Young inequality, we can estimate the coupling terms as follows,

(47) $$\begin{array}{ccc} \; & \; & |\Phi_{N}(∥\nabla U_{l_{j}}{∥}_{L^{\infty }\left(R^{3}\right)})\langle \left(u_{l_{j}}\cdot \nabla \right)\theta_{R}{\tilde{u}}_{l_{j}}-\left(m_{l_{j}}\cdot \nabla \right)\theta_{R}{\tilde{m}}_{l_{j}}-\left(\theta_{R}{\tilde{u}}_{l_{j}}\cdot \nabla \right)u_{l_{j}}\\ \; & \; & \;+\left(\theta_{R}{\tilde{m}}_{l_{j}}\cdot \nabla \right)m_{l_{j}}-\left(u_{l_{j}}\cdot \nabla \theta_{R}\right){\tilde{u}}_{l_{j}}+\left(m_{l_{j}}\cdot \nabla \theta_{R}\right){\tilde{m}}_{l_{j}},\theta_{R}{\tilde{u}}_{l_{j}}-\theta_{R}\nabla \times {\hat{q}}_{l_{j}}\rangle \\ \; & \; & \;+\Phi_{N}(∥\nabla U_{l_{j}}{∥}_{L^{\infty }\left(R^{3}\right)})\langle \left(u_{l_{j}}\cdot \nabla \right)\theta_{R}{\tilde{m}}_{l_{j}}-\left(m_{l_{j}}\cdot \nabla \right)\theta_{R}{\tilde{u}}_{l_{j}}-\left(\theta_{R}{\tilde{u}}_{l_{j}}\cdot \nabla \right)m_{l_{j}}\\ \; & \; & \;+\left(\theta_{R}{\tilde{m}}_{l_{j}}\cdot \nabla \right)u_{l_{j}}-\left(u_{l_{j}}\cdot \nabla \theta_{R}\right){\tilde{m}}_{l_{j}}+\left(m_{l_{j}}\cdot \nabla \theta_{R}\right){\tilde{u}}_{l_{j}},\theta_{R}{\tilde{m}}_{l_{j}}-\theta_{R}\nabla \times q_{l_{j}}\rangle |\\ \; & \; & \;+\Phi_{N}(∥\nabla U_{l_{j}}{∥}_{L^{\infty }\left(R^{3}\right)})|\langle \theta_{R}f,\theta_{R}{\tilde{m}}_{l_{j}}-\theta_{R}\nabla \times q_{l_{j}}\rangle |\\ \; & \; & \leq C∥\theta_{R}{\tilde{u}}_{l_{j}}{∥}_{0}^{2}+C∥\theta_{R}\nabla \times q_{l_{j}}{∥}_{0}+C∥\theta_{R}\nabla \times q_{l_{j}}{∥}_{0}^{2}+C∥\theta_{R}{\tilde{m}}_{l_{j}}{∥}_{0}^{2}+C{∥\theta_{R}f∥}_{0}^{2}\\ \; & \; & \;+C∥\theta_{R}\nabla \times {\hat{q}}_{l_{j}}{∥}_{0}+C{∥\theta_{R}\nabla \times {\hat{q}}_{l_{j}}∥}_{0}^{2}+\frac{C}{R^{2}}, \end{array}$$

where C>0 denotes constant independent of $l_{j},R\geq 1$. By Equations (45)–(47), we obtain

(48) $$\begin{array}{ccc} \; & \; & ∥\theta_{R}{\tilde{U}}_{l_{j}}-\theta_{R}\nabla \times Q_{l_{j}}{∥}_{0}^{2}\leq ∥\theta_{R}{\tilde{U}}_{l_{j}}{\left(0\right)∥}_{0}^{2}+C\int_{0}^{t}{∥\theta_{R}{\tilde{U}}_{l_{j}}\left(s\right)∥}_{0}^{2}ds\\ \; & \; & +C\int_{0}^{t}∥\theta_{R}\nabla \times Q_{l_{j}}{∥}_{0}^{2}ds+C\int_{0}^{t}∥\theta_{R}\nabla \times Q_{l_{j}}{\left(s\right)∥}_{0}ds+\frac{C}{R^{2}}+C\int_{0}^{t}{∥\theta_{R}f∥}_{0}^{2}ds,\; \end{array}$$

for all t∈[0,T], where C>0 is a constant independent of $l_{j},R\geq 1,t\in \left[0,T\right)$ but depends on *T*. As

$$\begin{array}{c} {\tilde{U}}_{l_{j}}\left(0\right)\to \nabla \times U_{0}\;\mathrm{in}\;H_{\sigma }^{\alpha -1},\;Q_{l_{j}}\to Q\;\mathrm{in}\;C\left(\left[0,T\right];H_{\sigma }^{\alpha }\right)\;\mathrm{as}\;l_{j}\to \infty , \end{array}$$

and

$$\begin{array}{c} \theta_{R}{\tilde{U}}_{l_{j}}\left(0\right)\to 0\;\mathrm{strongly}\;\mathrm{in}\;L^{2}\left(R^{3}\right),\;\;\theta_{R}\nabla \times Q_{l_{j}}\left(t\right)\to 0\;\mathrm{strongly}\;\mathrm{in}\;L^{2}\left(R^{3}\right)\;\mathrm{as}\;R\to \infty \end{array}$$

uniformly in $l_{j}$ and t∈[0,T]. It follows that $\int_{0}^{t}{∥\theta_{R}\nabla \times Q_{l_{j}}\left(s\right)∥}_{0}^{2}ds\to 0$ as R→∞. We also have $\int_{0}^{t}{∥\theta_{R}f∥}_{0}^{2}ds\to 0$ as R→∞ due to the definition of $\theta_{R}$ and (34). Due to Grönwall inequality, it follows from (48) that

(49) $$\begin{array}{c} ∥\theta_{R}{\tilde{U}}_{l_{j}}{∥}_{0}\to 0,\;as\;R\to \infty ,\;\mathrm{uniformly}\;\mathrm{in}\;l_{j}\;\mathrm{and}\;t\in \left[0,T\right]. \end{array}$$

On the other hand, by Lemma 3 and (34), there is a constant C>0 independent of $l_{j},R\geq 1$ and t∈[0,T] such that

(50) $$\begin{array}{c} ∥\theta_{R}{\tilde{U}}_{l_{j}}{\left(t\right)∥}_{\alpha -1}\leq C. \end{array}$$

By Equations (45) and (46) as well as the interpolation inequality ${∥h∥}_{\beta -1}\leq {C∥h∥}_{0}^{1-\lambda }{∥h∥}_{\alpha -1}^{\lambda },\forall h\in H^{\alpha -1}\left(R^{3}\right)$, for some constant *C* and $\lambda =\frac{\beta -1}{\alpha -1}$ we have

(51) $$\begin{array}{c} ∥\theta_{R}{\tilde{U}}_{l_{j}}{\left(t\right)∥}_{\beta -1}\to 0,\;\mathrm{as}\;R\to \infty ,\;\mathrm{uniformly}\;\mathrm{in}\;l_{j}\;\;t\in \left[0,T\right]. \end{array}$$

Applying (51), Lemma 3 and the identity $\nabla \times \left(\theta_{R}U_{l_{j}}\right)=\theta_{R}\left(\nabla \times U_{l_{j}}\right)+\left(\nabla \theta_{R}\right)\times U_{l_{j}}$, we obtain

(52) $$\begin{array}{c} ∥\nabla \times \left(\theta_{R}U_{l_{j}}\left(t\right)\right){∥}_{\beta -1}\to 0\;\mathrm{as}\;R\to \infty ,\;\mathrm{uniformly}\;\mathrm{in}\;l_{j}\;\mathrm{and}\;t\in \left[0,T\right]. \end{array}$$

Due to the facts

(53) $$\begin{array}{ccc} \sum_{i=1}^{3}\Delta^{-1}\partial_{j}\partial_{i}\left(\partial_{i}v_{k}-\partial_{k}v_{i}\right) & = & \partial_{j}v_{k}-\Delta^{-1}\partial_{j}\partial_{k}\left(\nabla \cdot v\right), \end{array}$$

(54) $$\begin{array}{ccc} \nabla \cdot (\theta_{R}U_{l_{j}}) & = & \theta_{R}\left(\nabla \cdot U_{l_{j}}\right)+\left(\nabla \theta_{R}\right)\cdot U_{l_{j}}, \end{array}$$

for $j,k=1,2,3,v=\left(v_{1},v_{2},v_{3}\right)\in H^{1}\left(R^{3}\right)$, we have

(55) $$\begin{array}{c} ∥\left(\nabla \theta_{R}\right)\cdot U_{l_{j}}{∥}_{\beta -1}\to 0,\;\mathrm{as}\;R\to \infty ,\;\mathrm{uniformly}\;\mathrm{in}\;l_{j}\;\mathrm{and}\;t\in \left[0,T\right]. \end{array}$$

The Riesz transform is continuous from $H^{s}\left(R^{3}\right)$ into itself for any *s* and Equations (52)–(55), we have

(56) $$\begin{array}{c} ∥\nabla \left(\theta_{R}U_{l_{j}}\left(t\right)\right){∥}_{\beta -1}\to 0,\;\mathrm{as}\;R\to \infty ,\;\;\mathrm{uniformly}\;\mathrm{in}\;l_{j}\;\mathrm{and}\;t\in \left[0,T\right]. \end{array}$$

By (39), we also have

(57) $$\begin{array}{c} \Phi_{N}(∥\nabla U_{l_{j}}{∥}_{L^{\infty }\left(R^{3}\right)})\to \Phi_{N}{(∥\nabla U∥}_{L^{\infty }\left(R^{3}\right)})\;\;\mathrm{strongly}\;\mathrm{in}\;C\left(\left[0,T\right]\right). \end{array}$$

Then, combining (37) and (57), we obtain

(58) $$\begin{array}{c} \frac{\partial U}{\partial t}+\Phi_{N}{(∥\nabla U∥}_{L^{\infty }\left(R^{3}\right)})\Pi B\left(U\right)=\frac{\partial Q}{\partial t} \end{array}$$

in the sense of distributions over $R^{3}\times \left[0,T\right]$, at $\omega^{*}$. We claim that

(59) $$\begin{array}{c} U\in C\left(\left[0,T\right];H_{\sigma }^{\beta }\right),\;\mathrm{at}\;\omega^{*}. \end{array}$$

Due to (37) and (58), we have $\frac{\partial }{\partial t}\left(U-Q\right)\in L^{\infty }\left(0,T;H_{\sigma }^{\alpha -1}\right).$ Because of the fact that $U_{0}\in H_{\sigma }^{\alpha }$ and $Q\in C(\left[0,T\right];H_{\sigma }^{\alpha })$, we obtain

(60) $$\begin{array}{c} U\in C(\left[0,T\right];H_{\sigma }^{\alpha -1}). \end{array}$$

As α−1>β−1, Equations (39) and (60) imply Equation (59).

Due to the occurrence of the cut-off function which plays a key role to obtain the uniformly boundedness and also brings the difficulty of uniqueness, we need to introduce a function ${\hat{\tau }}_{N,i}$ as follows

(61) $$\begin{array}{c} {\hat{\tau }}_{N,i}=\left\{\begin{array}{c} \inf\left\{0\leq t\leq T:∥\nabla U_{i}{∥}_{L^{\infty }\left(R^{3}\right)}\geq N\right\},\\ T,\;\mathrm{if}\;\mathrm{the}\;\mathrm{above}\;\mathrm{set}\;\{\cdots \}\;\mathrm{is}\;\mathrm{empty}, \end{array}\right.i=1,2, \end{array}$$

for the same fixed $\omega^{*}\in \Omega^{*}$. Assume that ${\hat{\tau }}_{N,1}\wedge {\hat{\tau }}_{N,2}>0$. Let $U_{1}$ and $U_{2}$ be two functions satisfying (58), (59), and $U_{1}\left(0\right)=U_{2}\left(0\right)=U_{0}$. It holds that

$$\begin{array}{c} \frac{\partial }{\partial t}\left(u_{1}-u_{2}\right)+\Pi \left(u_{1}\cdot \nabla \right)u_{1}-\Pi \left(u_{2}\cdot \nabla \right)u_{2}-\Pi \left(m_{1}\cdot \nabla \right)m_{1}+\Pi \left(m_{2}\cdot \nabla \right)m_{2}=0,\\ \frac{\partial }{\partial t}\left(m_{1}-m_{2}\right)+\Pi \left(u_{1}\cdot \nabla \right)m_{1}-\Pi \left(u_{2}\cdot \nabla \right)m_{2}-\Pi \left(m_{1}\cdot \nabla \right)u_{1}+\Pi \left(m_{2}\cdot \nabla \right)u_{2}=0 \end{array}$$

in the sense of distributions over $R^{3}\times \left[0,{\hat{\tau }}_{N,1}\wedge {\hat{\tau }}_{N,2}\right]$. By (4), (5) and the Young inequality, we have

$$\begin{array}{c} \;\;\;|\langle \Pi \left(u_{1}\cdot \nabla \right)u_{1}-\Pi \left(u_{2}\cdot \nabla \right)u_{2}-\Pi \left(m_{1}\cdot \nabla \right)m_{1}+\Pi \left(m_{2}\cdot \nabla \right)m_{2},u_{1}-u_{2}\rangle \\ \;\;\;+\langle \Pi \left(u_{1}\cdot \nabla \right)m_{1}-\Pi \left(u_{2}\cdot \nabla \right)m_{2}-\Pi \left(m_{1}\cdot \nabla \right)u_{1}+\Pi \left(m_{2}\cdot \nabla \right)u_{2},m_{1}-m_{2}\rangle |\\ \leq C(∥u_{1}-u_{2}{∥}_{0}^{2}+∥m_{1}-m_{2}{∥}_{0}^{2}+∥u_{1}-u_{2}{∥}_{0}∥m_{1}-m_{2}{∥}_{0})\leq C∥U_{1}-U_{2}{∥}_{0}^{2}. \end{array}$$

It follows that

(62) $$\begin{array}{c} ∥U_{1}-U_{2}{∥}_{0}^{2}\leq C\int_{0}^{t}{∥U_{1}-U_{2}∥}_{0}^{2}ds, \end{array}$$

for all $t\in [0,{\hat{\tau }}_{N,1}\wedge {\hat{\tau }}_{N,2}]$ and some constant *C*, which yields

(63) $$\begin{array}{c} U_{1}=U_{2},\;\mathrm{for}\;\mathrm{all}\;t\in \left[0,{\hat{\tau }}_{N,1}\wedge {\hat{\tau }}_{N,2}\right]. \end{array}$$

Thus, ${\hat{\tau }}_{N,1}={\hat{\tau }}_{N,2}$. If ${\hat{\tau }}_{N,1}\wedge {\hat{\tau }}_{N,2}=0$, then ${\hat{\tau }}_{N,1}={\hat{\tau }}_{N,2}=0$ is obvious.

Now for each $\omega \in \Omega^{*}$, ${\hat{\tau }}_{N}$ associated with a limit function *U* of a certain subsequence $U_{l_{j}}$ is uniquely determined, and *U* is unique on the interval $\left[0,{\hat{\tau }}_{N}\right]$.

Next, we need to show that ${\hat{\tau }}_{N}$ is a stopping time and $U(\cdot \wedge {\hat{\tau }}_{N})$ is $H_{\sigma }^{\alpha }$-valued progressively measurable.

**Proposition** **3.** *${\hat{\tau }}_{N}$ is a stopping time.*

**Proof.**  We need to show that the set $\left\{{\hat{\tau }}_{N}>t\right\}$ is $F_{t}$-measurable for 0≤t<T. We first claim that

(64) $$\begin{array}{ccc} \left\{{\hat{\tau }}_{N}>t\right\}\cap \Omega^{*}=\Omega^{*} & \cap & {⋃}_{L=1}^{\infty }{⋃}_{\nu =1}^{\infty }{⋂}_{k=1}^{\infty }{⋃}_{l=k}^{\infty }\left\{∥\nabla U_{l}{∥}_{C(\left[0,t\right];L^{\infty }\left(R^{3}\right))}\leq N-\frac{1}{\nu }\right\}\\ \; & \cap & \left\{∥U_{l}{∥}_{C(\left[0,t\right];H_{\sigma }^{\alpha })}\leq L\right\}. \end{array}$$

Let $\omega \in \left\{{\hat{\tau }}_{N}>t\right\}\cap \Omega^{*}$. Then, according to the above procedure, there exists a subsequence $\left\{U_{l_{j}}\right\}$ such that (34), (37)–(39) hold for some function *U*. Then, ${\hat{\tau }}_{N}$ can be defined in terms of the limit function *U*. As ${\hat{\tau }}_{N}>t$, it holds ${∥\nabla U∥}_{C(\left[0,t\right];L^{\infty }\left(R^{3}\right))}\leq N-\delta$, for some δ. Therefore, we have $∥\nabla U_{l_{j}}{∥}_{C(\left[0,t\right];L^{\infty }\left(R^{3}\right))}\leq N-\frac{\delta }{2}$ for all sufficiently large $l_{j}$. Then, ω belongs to the right-hand set.

Next, ω belongs to the right-hand set. As $\omega \in \Omega^{*}$, there exists a subsequence $\left\{U_{l_{j}}\right\}$ such that Equations (34) and (37)–(39) hold for some function *U* and ${\hat{\tau }}_{N}$ can be defined in terms of this limit function *U*. Simultaneously, there exists another subsequence ${\hat{U}}_{l_{j}}$, such that for some L≥1 and ν≥1

$$\begin{array}{c} ∥{\hat{U}}_{l_{j}}{∥}_{C(\left[0,t\right];H_{\sigma }^{\alpha })}\leq L,\;\;{∥\nabla {\hat{U}}_{l_{j}}∥}_{C(\left[0,t\right];L^{\infty }\left(R^{3}\right))}\leq N-\frac{1}{\nu }, \end{array}$$

for all $l_{j}$. Applying the above procedure on the interval [0,t], we can further extract a subsequence still denoted by ${\hat{U}}_{l_{j}}$ which satisfies (35), (37)–(39) hold for some function $\hat{U}$, which satisfies that

(65) $$\begin{array}{c} ∥\nabla \hat{U}{∥}_{C(\left[0,t\right];L^{\infty }\left(R^{3}\right))}\leq N-\frac{1}{\nu }. \end{array}$$

Since $\hat{U}\in C\left(\left[0,t\right];H_{\sigma }^{\beta }\right)$, $U\in C(\left[0,T\right];H_{\sigma }^{\beta })$ and repeating the above procedure on the uniqueness, we obtain

$$\begin{array}{c} \hat{U}=U\;\mathrm{on}\;\left[0,t\wedge {\hat{\tau }}_{N}\right]. \end{array}$$

Therefore, ${\hat{\tau }}_{N}>t$ follows from (65). Thus, $\omega \in \left\{{\hat{\tau }}_{N}>t\right\}\cap \Omega^{*}$. As the left-hand set is $F_{t}$-measurable for 0≤t<T. For t≥T, the left-hand set is empty, so it is $F_{t}$-measurable. Thus, ${\hat{\tau }}_{N}$ is a stopping time. □

**Proposition** **4.** *$U(\cdot \wedge {\hat{\tau }}_{N})$ is $H_{\sigma }^{\alpha }$-valued progressively measurable.*

**Proof.**  For each $\omega \in \Omega^{*}$, $U\left(\cdot \wedge {\hat{\tau }}_{N}\right)\in C\left(\left[0,T\right];H_{\sigma }^{\beta }\right)\cap L^{\infty }\left(0,T;H_{\sigma }^{\alpha }\right)$. Thus, $U\left(t\wedge {\hat{\tau }}_{N}\right)\in H_{\sigma }^{\alpha }$. Meanwhile, every Borel subset of $H_{\sigma }^{\alpha }$ is also a Borel subset of $H_{\sigma }^{\beta }$; therefore, every Borel subset of $C(\left[0,T\right];H_{\sigma }^{\alpha })$ is also a Borel subset of $C(\left[0,T\right];H_{\sigma }^{\beta })$. Using $\theta_{R}$, we define $\phi_{R}\left(x\right)=1-\theta_{R}\left(x\right)$, for all *x*. Then, for all $\omega \in \Omega^{*}$, we have

$$\begin{array}{c} \phi_{R}U\left(\cdot \wedge {\hat{\tau }}_{N}\right)\to U\left(\cdot \wedge {\hat{\tau }}_{N}\right)\;\mathrm{in}\;C\left(\left[0,T\right];H^{\beta }\left(R^{3}\right)\right), \end{array}$$

as R→∞, for every 0<T<∞. As the continuity of time of *U*, it is enough to show the measurability of $\phi_{R}U\left(\cdot \wedge {\hat{\tau }}_{N}\right)$ for each *R* in $B(C\left(\left[0,T\right];H^{\beta }\left(R^{3}\right)\right))$. Fixed any 0<t<∞ and let $\bar{B_{r}\left(p\right)}$ be the closure of an open ball $B_{r}\left(p\right)$ with radius r>0 and center *p* in $C(\left[0,T\right];H^{\beta }\left(R^{3}\right))$. We first claim that

(66) $$\begin{array}{ccc} \left\{\phi_{R}U\left(\cdot \wedge {\hat{\tau }}_{N}\right)\in \bar{B_{r}\left(p\right)}\right\}\cap \Omega^{*}=\Omega^{*} & \cap & {⋃}_{L=1}^{\infty }{⋃}_{\nu =1}^{\infty }{⋂}_{k=1}^{\infty }{⋃}_{l=k}^{\infty }\left\{\phi_{R}U_{l}\left(\cdot \wedge {\hat{\tau }}_{N}\right)\in B_{r+\frac{1}{\nu }}\left(p\right)\right\}\\ \; & \cap & \{∥U_{l}\left(\cdot \wedge {\hat{\tau }}_{N}\right){∥}_{C(\left[0,T\right];H_{\sigma }^{\alpha }\left(R^{3}\right))}\leq L\}. \end{array}$$

Let $\omega \in \left\{\phi_{R}U\left(\cdot \wedge {\hat{\tau }}_{N}\right)\in \bar{B_{r}\left(p\right)}\right\}\cap \Omega^{*}$. Then, according to the above procedure, there exists a subsequence $\left\{U_{l_{j}}\right\}$ such that Equations (34) and (37)–(39) hold. By Equation (37), for any ν≥1, $\phi_{R}U_{l_{j}}\left(\cdot \wedge {\hat{\tau }}_{N}\right)\in B_{r+\frac{1}{\nu }}\left(p\right)$, for all sufficiently large $l_{j}$. Thus, ω belongs to the right-hand set.

Next, ω belongs to the right-hand set. Since $\omega \in \Omega^{*}$, $U(\cdot \wedge {\hat{\tau }}_{N}\left(\omega \right))$ is well-defined. At the same time, for some L≥1 such that for each ν≥1, there exists a certain subsequence $\left\{U_{l_{j}}\right\}$ such that (34), (37)–(39) hold for some function $\tilde{U}$ with *T* replaced by $t\wedge {\hat{\tau }}_{N}\left(\omega \right)$. Let

$$\begin{array}{c} {\tilde{\tau }}_{N}=\left\{\begin{array}{c} \inf\left\{0\leq s\leq t\wedge {\hat{\tau }}_{N}\left(\omega \right):{∥\nabla \tilde{U}∥}_{L^{\infty }\left(R^{3}\right)}\geq N\right\},\\ t\wedge {\hat{\tau }}_{N}\left(\omega \right),\;\mathrm{if}\;\mathrm{the}\;\mathrm{above}\;\mathrm{set}\;\left\{\cdots \right\}\;\mathrm{is}\;\mathrm{empty}. \end{array}\right. \end{array}$$

Suppose ${\tilde{\tau }}_{N}>0$. Then, *U* and $\tilde{U}$ satisfy

$$\begin{array}{c} \frac{\partial }{\partial t}\left(U-\tilde{U}\right)+\Pi B\left(U\right)-\Pi B\left(\tilde{U}\right)=0 \end{array}$$

in the sense of distributions over $R^{3}\times \left(0,{\tilde{\tau }}_{N}\right)$. It follows that $U=\tilde{U}$ on $\left[0,{\tilde{\tau }}_{N}\right]$; therefore, ${\tilde{\tau }}_{N}=t\wedge {\hat{\tau }}_{N}\left(\omega \right)$. Then $U=\tilde{U}$ on $\left[0,t\wedge {\hat{\tau }}_{N}\left(\omega \right)\right]$. As $\phi_{R}U_{l_{j}}\left(\cdot \right)\to \phi_{R}\tilde{U}\left(\cdot \right)$ in $C(\left[0,t\wedge {\hat{\tau }}_{N}\left(\omega \right)\right];H^{\beta }\left(R^{3}\right))$; therefore, $\phi_{R}\tilde{U}\left(\cdot \wedge {\hat{\tau }}_{N}\left(\omega \right)\right)\in \bar{B_{r+\frac{1}{\nu }}\left(p\right)}$. Thus,

$$\begin{array}{c} \phi_{R}U\left(\cdot \wedge {\hat{\tau }}_{N}\left(\omega \right)\right)\in \bar{B_{r+\frac{1}{\nu }}\left(p\right)} \end{array}$$

holds for all ν≥1. Then, ω belongs to the left-hand set, and Equation (66) is valid.

As ${\hat{\tau }}_{N}$ is a stopping time, the right-hand set belongs to $F_{t}$. Consequently, the map from [0,t]×Ω into $H^{\beta }\left(R^{3}\right)$ defined by $\left(s,\omega \right)\mapsto \phi_{R}U\left(s\wedge {\hat{\tau }}_{N}\right)$ is $B\left(\left[0,t\right]\right)\times F_{t}$-measurable. □

To improve the time regularity of $U(\cdot \wedge {\hat{\tau }}_{N})$, we need the next lemma which will be used in Proposition 5.

**Lemma** **4.** *It holds that*

(67) $$\begin{array}{c} E(∥U\left(t\wedge {\hat{\tau }}_{N}\right){∥}_{L^{\infty }\left(0,T;H_{\sigma }^{\alpha }\right)}^{2})\leq C_{N,T} \end{array}$$

*where $C_{N,T}$ is the same constant as in (34).*

**Proof.**  Choosing any constant K>0. We first claim that for each $\omega \in \Omega^{*}$,

(68) $$\begin{array}{c} ∥U\left(\cdot \wedge {\hat{\tau }}_{N}\left(\omega \right)\right){∥}_{L^{\infty }\left(0,T;H_{\sigma }^{\alpha }\right)}\wedge K\leq \lim_{\bar{l\to \infty }}{∥U_{l}\left(\cdot \right)∥}_{L^{\infty }\left(0,T;H_{\sigma }^{\alpha }\right)}\wedge K. \end{array}$$

For any fixed $\omega \in \Omega^{*}$. If the right-hand side is equal to *K*, then (68) holds. Suppose

$$\begin{array}{c} \lim_{\bar{l\to \infty }}{∥U_{l}\left(\cdot \right)∥}_{L^{\infty }\left(0,T;H_{\sigma }^{\alpha }\right)}\wedge K=\gamma <K. \end{array}$$

Then, there exists a subsequence $\left\{U_{l_{j}}\right\}$ such that $\lim_{l\to \infty }{∥U_{l_{j}}\left(\cdot \right)∥}_{L^{\infty }\left(0,T;H_{\sigma }^{\alpha }\right)}=\gamma$. By repetition of the above procedure, we can further extract a subsequence still denoted by $\left\{U_{l_{j}}\right\}$ such that

(69) $$\begin{array}{c} U_{l_{j}}\to \tilde{U}\;\mathrm{weak}\;\mathrm{star}\;\mathrm{in}\;L^{\infty }\left(0,T;H_{\sigma }^{\alpha }\right), \end{array}$$

(70) $$\begin{array}{c} \tilde{U}\in C\left(\left[0,T\right];H_{\sigma }^{\beta }\right)\cap L^{\infty }\left(0,T;H_{\sigma }^{\alpha }\right), \end{array}$$

(71) $$\begin{array}{c} U\left(\cdot \wedge {\hat{\tau }}_{N}\left(\omega \right)\right)\equiv \tilde{U}\left(\cdot \wedge {\hat{\tau }}_{N}\left(\omega \right)\right), \end{array}$$

for some function $\tilde{U}$. Combining Equations (69) and (71), we have

$$\begin{array}{c} ∥U\left(\cdot \wedge {\hat{\tau }}_{N}\left(\omega \right)\right){∥}_{L^{\infty }\left(0,T;H_{\sigma }^{\alpha }\right)}=∥\tilde{U}\left(\cdot \wedge {\hat{\tau }}_{N}\left(\omega \right)\right){∥}_{L^{\infty }\left(0,T;H_{\sigma }^{\alpha }\right)}\leq {∥\tilde{U}\left(\cdot \right)∥}_{L^{\infty }\left(0,T;H_{\sigma }^{\alpha }\right)}\leq \gamma . \end{array}$$

Therefore, we obtain (68). Next, by applying the Fatou Lemma and (34), which yields

$$\begin{array}{c} E(∥U\left(\cdot \wedge {\hat{\tau }}_{N}\left(\omega \right)\right){∥}_{L^{\infty }\left(0,T;H_{\sigma }^{\alpha }\right)}^{2}\wedge K)\leq \lim_{\bar{l\to \infty }}E(∥U_{l}\left(\cdot \right){∥}_{L^{\infty }\left(0,T;H_{\sigma }^{\alpha }\right)}\wedge K)\leq C_{N,T}, \end{array}$$

for all K>0. By passing K→∞, we obtain (67). □

Next, we improve the time regularity of $U(\cdot \wedge {\hat{\tau }}_{N})$.

**Proposition** **5.** *It holds that $U\left(\cdot \wedge {\hat{\tau }}_{N}\right)\in C\left(\left[0,T\right];H_{\sigma }^{\alpha }\right)$, for almost all ω.*

**Proof.**  Let $\rho_{\delta }$ be the Friedrichs mollifier with respect to the space variable. It holds that

$$\begin{array}{ccc} u\left(t\wedge {\hat{\tau }}_{N}\right)*\rho_{\delta } & = & u_{0}*\rho_{\delta }-\int_{0}^{t\wedge {\hat{\tau }}_{N}}\Pi \left(\left(u\left(s\right)\cdot \nabla \right)u\left(s\right)-\left(m\left(s\right)\cdot \nabla \right)m\left(s\right)\right)*\rho_{\delta }ds\\ & & +\Pi \sum_{j=1}^{\infty }\int_{0}^{t\wedge {\hat{\tau }}_{N}}g_{1,j}\left(s\right)*\rho_{\delta }d\beta_{j}^{1}\left(s\right),\\ m\left(t\wedge {\hat{\tau }}_{N}\right)*\rho_{\delta } & = & m_{0}*\rho_{\delta }-\int_{0}^{t\wedge {\hat{\tau }}_{N}}\Pi \left(\left(u\left(s\right)\cdot \nabla \right)m\left(s\right)-\left(m\left(s\right)\cdot \nabla \right)u\left(s\right)\right)*\rho_{\delta }ds\\ & & +\Pi \sum_{j=1}^{\infty }\int_{0}^{t\wedge {\hat{\tau }}_{N}}g_{2,j}\left(s\right)*\rho_{\delta }d\beta_{j}^{2}\left(s\right), \end{array}$$

for all 0≤t<∞ and δ>0, for each $\omega \in \Omega^{*}$. By applying the Itô formula to the functions $∥u\left(t\wedge {\hat{\tau }}_{N}\right)*\rho_{\delta }{∥}_{\alpha }^{2}$ and $∥m\left(t\wedge {\hat{\tau }}_{N}\right)*\rho_{\delta }{∥}_{\alpha }^{2}$, we have

$$\begin{array}{cc} & ∥u\left(t_{2}\wedge {\hat{\tau }}_{N}\right)*\rho_{\delta }{∥}_{\alpha }^{2}-{∥u\left(t_{1}\wedge {\hat{\tau }}_{N}\right)*\rho_{\delta }∥}_{\alpha }^{2}\\ = & -2\int_{t_{1}}^{t_{2}}{\langle \left(\left(u\left(s\right)\cdot \nabla \right)u\left(s\right)-\left(m\left(s\right)\cdot \nabla \right)m\left(s\right)\right)*\rho_{\delta },u\left(s\right)*\rho_{\delta }\rangle }_{\alpha }I_{\left[0,{\hat{\tau }}_{N}\right]}\left(s\right)ds\\ & +2\sum_{j=1}^{\infty }\int_{t_{1}}^{t_{2}}{\langle g_{1,j}\left(s\right)*\rho_{\delta },u\left(s\right)*\rho_{\delta }\rangle }_{\alpha }I_{\left[0,{\hat{\tau }}_{N}\right]}\left(s\right)d\beta_{j}^{1}\left(s\right)\\ & +\sum_{j=1}^{\infty }\int_{t_{1}}^{t_{2}}{∥g_{1,j}\left(s\right)*\rho_{\delta }∥}_{\alpha }^{2}I_{\left[0,{\hat{\tau }}_{N}\right]}\left(s\right)ds,\\ & ∥m\left(t_{2}\wedge {\hat{\tau }}_{N}\right)*\rho_{\delta }{∥}_{\alpha }^{2}-{∥m\left(t_{1}\wedge {\hat{\tau }}_{N}\right)*\rho_{\delta }∥}_{\alpha }^{2}\\ = & -2\int_{t_{1}}^{t_{2}}{\langle \left(\left(u\left(s\right)\cdot \nabla \right)m\left(s\right)-\left(m\left(s\right)\cdot \nabla \right)u\left(s\right)\right)*\rho_{\delta },m\left(s\right)*\rho_{\delta }\rangle }_{\alpha }I_{\left[0,{\hat{\tau }}_{N}\right]}\left(s\right)ds\\ & +2\sum_{j=1}^{\infty }\int_{t_{1}}^{t_{2}}{\langle g_{2,j}\left(s\right)*\rho_{\delta },m\left(s\right)*\rho_{\delta }\rangle }_{\alpha }I_{\left[0,{\hat{\tau }}_{N}\right]}\left(s\right)d\beta_{j}^{2}\left(s\right)\\ & +\sum_{j=1}^{\infty }\int_{t_{1}}^{t_{2}}{∥g_{2,j}\left(s\right)*\rho_{\delta }∥}_{\alpha }^{2}I_{\left[0,{\hat{\tau }}_{N}\right]}\left(s\right)ds, \end{array}$$

for all $0\leq t_{1}<t_{2}<\infty$, $\delta =\delta_{n}=\frac{1}{n},n=1,2,\cdots ,$ for each $\omega \in \tilde{\Omega }$ with $P(\Omega^{*}\backslash \tilde{\Omega })=0$.

By Equations (14)–(16), we have

(72) $$\begin{array}{ccc} \; & \; & \underset{\delta_{n}\to 0}{lim sup}|\int_{t_{1}}^{t_{2}}{\langle \left(\left(u\left(s\right)\cdot \nabla \right)u\left(s\right)-\left(m\left(s\right)\cdot \nabla \right)m\left(s\right)\right)*\rho_{\delta },u\left(s\right)*\rho_{\delta }\rangle }_{\alpha }I_{\left[0,{\hat{\tau }}_{N}\right]}\left(s\right)ds|\\ \; & \leq & C_{N}\int_{t_{1}}^{t_{2}}{(∥u\left(s\right)∥}_{\alpha }^{2}+{∥m\left(s\right)∥}_{\alpha }^{2})I_{\left[0,{\hat{\tau }}_{N}\right]}\left(s\right)ds, \end{array}$$

(73) $$\begin{array}{ccc} \; & \; & \underset{\delta_{n}\to 0}{lim sup}|\int_{t_{1}}^{t_{2}}{\langle \left(\left(u\left(s\right)\cdot \nabla \right)m\left(s\right)-\left(m\left(s\right)\cdot \nabla \right)u\left(s\right)\right)*\rho_{\delta },m\left(s\right)*\rho_{\delta }\rangle }_{\alpha }I_{\left[0,{\hat{\tau }}_{N}\right]}\left(s\right)ds|\\ \; & \leq & C_{N}\int_{t_{1}}^{t_{2}}{(∥u\left(s\right)∥}_{\alpha }^{2}+{∥m\left(s\right)∥}_{\alpha }^{2})I_{\left[0,{\hat{\tau }}_{N}\right]}\left(s\right)ds, \end{array}$$

for all $0\leq t_{1}<t_{2}<\infty$ and some constant $C_{N}>0$. By the Burkholder–Davis–Gundy inequality, the identity ${\langle h*\rho_{\delta },g*\rho_{\delta }\rangle }_{\alpha }={\langle h*\rho_{\delta }*\rho_{\delta },g\rangle }_{\alpha }$ and Lemma 4, we have

$$\begin{array}{c} E\left(\sup_{0\leq t\leq T}|\sum_{j=1}^{\infty }\int_{0}^{t}\left[{\langle g_{1,j}\left(s\right)*\rho_{\delta },u\left(s\right)*\rho_{\delta }\rangle }_{\alpha }I_{\left[0,{\hat{\tau }}_{N}\right]}\left(s\right)-{\langle g_{1,j}\left(s\right),u\left(s\right)\rangle }_{\alpha }I_{\left[0,{\hat{\tau }}_{N}\right]}\left(s\right)\right]d\beta_{j}^{1}\left(s\right)|\right)\\ \leq CE\left(\sup_{0\leq t\leq T}∥u\left(t\wedge {\hat{\tau }}_{N}\right){∥}_{\alpha }(\int_{0}^{T}∥g_{1,j}\left(s\right)*\rho_{\delta }*\rho_{\delta }-g_{1,j}\left(s\right){{∥}_{\alpha }^{2}ds)}^{\frac{1}{2}}\right)\to 0,\;\mathrm{as}\;\delta \to 0. \end{array}$$

Then, there is a subsequence of $\left\{\delta_{n}\right\}$ still denoted by $\left\{\delta_{n}\right\}$ and a set $\hat{\Omega }\subset \tilde{\Omega }$ such that $P(\tilde{\Omega }\backslash \hat{\Omega })=0$ and

(74) $$\begin{array}{ccc} \; & \; & \sum_{j=1}^{\infty }\int_{0}^{\left(\cdot \right)}{\langle g_{1,j}\left(s\right)*\rho_{\delta },u\left(s\right)*\rho_{\delta }\rangle }_{\alpha }I_{\left[0,{\hat{\tau }}_{N}\right]})\left(s\right)d\beta_{j}^{1}\left(s\right)\\ \; & \; & \to \sum_{j=1}^{\infty }\int_{0}^{\left(\cdot \right)}{\langle g_{1,j}\left(s\right),u\left(s\right)\rangle }_{\alpha }I_{\left[0,{\hat{\tau }}_{N}\right]}\left(s\right)d\beta_{j}^{1}\left(s\right) \end{array}$$

in C([0,T]), as $\delta_{n}\to 0$. By the similar estimate, we also have

(75) $$\begin{array}{ccc} \; & \; & \sum_{j=1}^{\infty }\int_{0}^{\left(\cdot \right)}{\langle g_{2,j}\left(s\right)*\rho_{\delta },m\left(s\right)*\rho_{\delta }\rangle }_{\alpha }I_{\left[0,{\hat{\tau }}_{N}\right]}\left(s\right)d\beta_{j}^{2}\left(s\right)\\ \; & \; & \to \sum_{j=1}^{\infty }\int_{0}^{\left(\cdot \right)}{\langle g_{2,j}\left(s\right),m\left(s\right)\rangle }_{\alpha }I_{\left[0,{\hat{\tau }}_{N}\right]}\left(s\right)d\beta_{j}^{2}\left(s\right) \end{array}$$

in C([0,T]), as $\delta_{n}\to 0$.

Combining Equations (72)–(75) and $U\left(t\wedge {\hat{\tau }}_{N}\right)\in H_{\sigma }^{\alpha }$, for all 0≤t<∞ and let $\delta_{n}\to 0$, we obtain

(76) $$\begin{array}{ccc} \; & \; & |∥U\left(t_{2}\wedge {\hat{\tau }}_{N}\right){∥}_{\alpha }^{2}-∥U\left(t_{1}\wedge {\hat{\tau }}_{N}\right){∥}_{\alpha }^{2}|\\ \; & \; & \leq C_{N}\int_{t_{1}}^{t_{2}}{∥U\left(s\right)∥}_{\alpha }^{2}I_{\left[0,{\hat{\tau }}_{N}\right]}\left(s\right)ds+|\sum_{j=1}^{\infty }\int_{t_{1}}^{t_{2}}{\langle g_{1,j}\left(s\right),u\left(s\right)\rangle }_{\alpha }I_{\left[0,{\hat{\tau }}_{N}\right]}\left(s\right)d\beta_{j}^{1}\left(s\right)|\\ \; & \; & +|\sum_{j=1}^{\infty }\int_{t_{1}}^{t_{2}}{\langle g_{2,j}\left(s\right),m\left(s\right)\rangle }_{\alpha }I_{\left[0,{\hat{\tau }}_{N}\right]}\left(s\right)d\beta_{j}^{2}\left(s\right)|+\sum_{j=1}^{\infty }\int_{t_{1}}^{t_{2}}{∥G_{j}\left(s\right)∥}_{\alpha }^{2}I_{\left[0,{\hat{\tau }}_{N}\right]}\left(s\right)ds, \end{array}$$

for all 0≤t<∞ and $\omega \in \hat{\Omega }$. Since $U\left(\cdot \wedge {\hat{\tau }}_{N}\right)\in C\left(\left[0,T\right];H_{\sigma }^{\beta }\right)\cap L^{\infty }\left(0,T;H_{\sigma }^{\alpha }\right)$ for each $\omega \in \hat{\Omega }$, we obtain the continuity of the time following (76). □

Up to now, we obtain the existence of local smooth of (1) for any fixed N≥1 and T>0, i.e., there exist a stopping time ${\hat{\tau }}_{N}$ and a function *U* with the following properties.

(1) $U\left(t\wedge {\hat{\tau }}_{N}\right)\in C\left(\left[0,T\right];H_{\sigma }^{\alpha }\right)$ for almost all ω and it is $H_{\sigma }^{\alpha }$-valued progressively measurable.

(2) It holds that

$$\begin{array}{c} U\left(t\wedge {\hat{\tau }}_{N}\right)=U_{0}-\int_{0}^{t}\Pi B\left(U\right)I_{\left[0,{\hat{\tau }}_{N}\right]}ds+\Pi \sum_{j=1}^{\infty }\int_{0}^{t}G_{j}\left(s\right)I_{\left[0,{\hat{\tau }}_{N}\right]}dW_{j}\left(s\right), \end{array}$$

for all 0≤t<∞ and almost all ω.

(3) For almost all ω,

$$\begin{array}{c} {\hat{\tau }}_{N}=\left\{\begin{array}{c} \inf\left\{0\leq t\leq {T:∥\nabla U∥}_{L^{\infty }\left(R^{3}\right)}\geq N\right\},\\ T,\;\mathrm{if}\;\mathrm{the}\;\mathrm{above}\;\mathrm{set}\;\{\cdots \}\;\mathrm{is}\;\mathrm{empty}. \end{array}\right. \end{array}$$

**Step 2.** Extend the existence interval by passing the limit T→∞ for any fixed N≥1. 

Let $U_{T}$ be the solution obtained with T=1,2,⋯. ${\hat{\tau }}_{N,T}$ is a stopping time defined by

$$\begin{array}{c} {\hat{\tau }}_{N,T}=\left\{\begin{array}{c} \inf\left\{0\leq t\leq T:∥\nabla U_{T}{∥}_{L^{\infty }\left(R^{3}\right)}\geq N\right\},\\ T,\;\mathrm{if}\;\mathrm{the}\;\mathrm{above}\;\mathrm{set}\;\{\cdots \}\;\mathrm{is}\;\mathrm{empty}. \end{array}\right. \end{array}$$

There exists some subset $\Omega_{N}$ such that $P(\Omega \backslash \Omega_{N})=0$ and $\left(U_{T},{\hat{\tau }}_{N,T}\right)$ satisfies the above properties (1)–(3) ∀T=1,2,⋯ and $\omega \in \Omega_{N}$. It is easy to check that for $T_{1}<T_{2}$ and each $\omega \in \Omega_{N}$,

$$\begin{array}{c} U_{T_{1}}\left(t\right)=U_{T_{2}}\left(t\right),\;\forall t\in \left[0,{\hat{\tau }}_{N,T_{1}}\wedge {\hat{\tau }}_{N,T_{2}}\right], \end{array}$$

then, we obtain

$$\begin{array}{c} {\hat{\tau }}_{N,T_{1}}\leq {\hat{\tau }}_{N,T_{2}},\;\;U_{T_{1}}\left(\cdot \wedge {\hat{\tau }}_{N,T_{1}}\right)=U_{T_{2}}\left(\cdot \wedge {\hat{\tau }}_{N,T_{2}}\right),\;\forall \omega \in \Omega_{N}. \end{array}$$

If not, assume that there exists $\omega \in \Omega_{N}$, such that ${\hat{\tau }}_{N,T_{1}}\left(\omega \right)>{\hat{\tau }}_{N,T_{2}}\left(\omega \right)$ and $U_{T_{1}}\left(t\right)=U_{T_{2}}\left(t\right)$, for all $t\in [0,{\hat{\tau }}_{N,T_{2}}]$. By the definition of ${\hat{\tau }}_{N,T_{1}}$ and ${\hat{\tau }}_{N,T_{2}}$, we have $∥\nabla U_{T_{1}}\left({\hat{\tau }}_{N,T_{2}}\right)∥=∥\nabla U_{T_{2}}\left({\hat{\tau }}_{N,T_{2}}\right)∥=N$, it contradicts with $∥\nabla U_{T_{1}}\left({\hat{\tau }}_{N,T_{2}}\right)∥<N$.

Thus, we can define

$$\begin{array}{c} {\tilde{\tau }}_{N}=\lim_{T\to \infty }{\hat{\tau }}_{N,T},\;U\left(t\wedge {\tilde{\tau }}_{N}\right)=\lim_{T\to \infty }U_{T}\left(t\wedge {\hat{\tau }}_{N,T}\right),\;\forall t\in \left[0,+\infty \right). \end{array}$$

If ${\tilde{\tau }}_{N}<\infty$, then there is some $T^{*}$ such that ${\hat{\tau }}_{N,T^{*}}<T^{*}$ and $\forall T>T^{*}$,

$$\begin{array}{c} {\hat{\tau }}_{N,T^{*}}={\hat{\tau }}_{N,T}={\tilde{\tau }}_{N},\;U_{T^{*}}\left(t\wedge {\hat{\tau }}_{N,T^{*}}\right)=U_{T}\left(t\wedge {\hat{\tau }}_{N,T}\right)=U\left(t\wedge {\tilde{\tau }}_{N}\right),\;\forall t\in \left[0,+\infty \right). \end{array}$$

Thus

$$\begin{array}{c} {∥\nabla U\left(t\right)∥}_{L^{\infty }\left(R^{3}\right)}<N,\;\mathrm{if}\;t<{\tilde{\tau }}_{N};\;{∥\nabla U\left({\tilde{\tau }}_{N}\right)∥}_{L^{\infty }\left(R^{3}\right)}=N. \end{array}$$

If ${\tilde{\tau }}_{N}=\infty$, it holds that ∀T=1,2,⋯,

$$\begin{array}{c} {\hat{\tau }}_{N,T}={T,\;∥\nabla U\left(t\right)∥}_{L^{\infty }\left(R^{3}\right)}=\lim_{T\to \infty }{∥\nabla U_{T}\left(t\wedge {\hat{\tau }}_{N,T}\right)∥}_{L^{\infty }\left(R^{3}\right)}<N,\;\forall \;0\leq t<\infty . \end{array}$$

Therefore, ${\tilde{\tau }}_{N}$ (defined above) can be written as follows,

(77) $$\begin{array}{c} {\tilde{\tau }}_{N}=\left\{\begin{array}{c} \inf\left\{0\leq {t<\infty :∥\nabla U∥}_{L^{\infty }\left(R^{3}\right)}\geq N\right\},\\ \infty ,\;\mathrm{if}\;\mathrm{the}\;\mathrm{above}\;\mathrm{set}\;\{\cdots \}\;\mathrm{is}\;\mathrm{empty}. \end{array}\right. \end{array}$$

We also have $U\left(\cdot \wedge {\tilde{\tau }}_{N}\right)\in C\left(\left[0,\infty \right);H_{\sigma }^{\alpha }\right)$ for each $\omega \in \Omega_{N}$ and satisfies

(78) $$\begin{array}{c} U\left(t\wedge {\tilde{\tau }}_{N}\right)=U_{0}-\int_{0}^{t}\Pi B\left(U\right)I_{\left[0,{\tilde{\tau }}_{N}\right]}\left(s\right)ds+\Pi \sum_{j=1}^{\infty }\int_{0}^{t}G_{j}\left(s\right)I_{\left[0,{\tilde{\tau }}_{N}\right]}\left(s\right)dW_{j}\left(s\right). \end{array}$$

**Step 3.** Establish the maximal time of existence of local smooth solution. 

Next, we pass N→∞ to obtain the maximal time of existence of the local smooth solution. For each N=1,2,⋯, let $U_{N}$ be the solution obtained in step 2 and ${\tilde{\tau }}_{N}$ be the stopping time associated with $U_{N}$ by (78). Let $\Omega_{0}=\overset{\infty }{\underset{N=1}{⋂}}\Omega_{N}$, where $\Omega_{N}$ be the same as in step 2 for each *N*.

For any $N_{1}<N_{2}$, it is easy to check that for all 0≤t<∞ and each $\omega \in \Omega_{0}$,

$$\begin{array}{c} U_{N_{1}}\left(t\wedge {\tilde{\tau }}_{N_{1}}\wedge {\tilde{\tau }}_{N_{2}}\right)=U_{N_{2}}\left(t\wedge {\tilde{\tau }}_{N_{1}}\wedge {\tilde{\tau }}_{N_{2}}\right), \end{array}$$

then, we have

$$\begin{array}{c} {\tilde{\tau }}_{N_{1}}\leq {\tilde{\tau }}_{N_{2}},\;if\;N_{1}<N_{2},\;and\;U_{N_{1}}\left(t\wedge {\tilde{\tau }}_{N_{1}}\right)=U_{N_{2}}\left(t\wedge {\tilde{\tau }}_{N_{1}}\right),\;\forall t\in \left[0,+\infty \right). \end{array}$$

If not, assume that $\exists \;\omega \in \Omega_{0}$ such that ${\tilde{\tau }}_{N_{1}}>{\tilde{\tau }}_{N_{2}}$, if $N_{1}<N_{2}$, then $U_{N_{1}}\left(t\wedge {\tilde{\tau }}_{N_{2}}\right)=U_{N_{2}}\left(t\wedge {\tilde{\tau }}_{N_{2}}\right)$ for all 0≤t<∞, it follows that $∥\nabla U_{N_{2}}\left({\tilde{\tau }}_{N_{2}}\right){∥}_{L^{\infty }\left(R^{3}\right)}={∥\nabla U_{N_{1}}\left({\tilde{\tau }}_{N_{2}}\right)∥}_{L^{\infty }\left(R^{3}\right)}$. By the definition of ${\tilde{\tau }}_{N_{1}}$ and ${\tilde{\tau }}_{N_{2}}$, we have $∥\nabla U_{N_{1}}\left({\tilde{\tau }}_{N_{1}}\right){∥}_{L^{\infty }\left(R^{3}\right)}=N_{1}$, $∥\nabla U_{N_{2}}\left({\tilde{\tau }}_{N_{2}}\right){∥}_{L^{\infty }\left(R^{3}\right)}=N_{2}$ and $∥\nabla U_{N_{1}}\left({\tilde{\tau }}_{N_{2}}\right){∥}_{L^{\infty }\left(R^{3}\right)}<N_{1}$, this contradicts with $N_{1}<N_{2}$.

Then, we can define

$$\begin{array}{c} \tau =\lim_{N\to \infty }{\tilde{\tau }}_{N},\;U\left(t\right)=\lim_{N\to \infty }U_{N}\left(t\wedge {\tilde{\tau }}_{N}\right),\;\forall \;0\leq t<\tau ,\forall \;\omega \in \Omega_{0}. \end{array}$$

If $t<{\tilde{\tau }}_{N^{*}}$, for some $N^{*}\geq 1$, at some $\omega^{*}\in \Omega_{0}$, we infer that

$$\begin{array}{c} U_{N^{*}}\left(t\wedge {\tilde{\tau }}_{N^{*}}\right)=U_{N^{*}}\left(t\wedge {\tilde{\tau }}_{N^{*}}\wedge {\tilde{\tau }}_{N}\right)=U_{N}\left(t\wedge {\tilde{\tau }}_{N^{*}}\wedge {\tilde{\tau }}_{N}\right)=U_{N}\left(t\wedge {\tilde{\tau }}_{N}\right)=U\left(t\right), \end{array}$$

for all $N\geq N^{*}$ and

(79) $$\begin{array}{c} {∥\nabla U\left(t\right)∥}_{L^{\infty }\left(R^{3}\right)}<N^{*},\;at\;\omega^{*}. \end{array}$$

On the other hand, if ${\tilde{\tau }}_{N^{*}}<\infty$, at $\omega^{*}$,

(80) $$\begin{array}{c} U_{N^{*}}\left({\tilde{\tau }}_{N^{*}}\right)=U_{N}\left({\tilde{\tau }}_{N^{*}}\right)=U\left({\tilde{\tau }}_{N^{*}}\right),\;\forall \;N\geq N^{*}. \end{array}$$

We also have $U\left(\cdot \right)\in C(\left[0,\tau \right);H_{\sigma }^{\alpha })$, for each $\omega \in \Omega_{0}$.

We set U(t)=0, if t>τ. Then, U(·) is right continuous on [0,∞) and $F_{t}$-measurable for each 0≤t<∞. Therefore, it is $H_{\sigma }^{\alpha }$-valued progressively measurable. By Equations (78) and (79), we define

$$\begin{array}{c} \tau_{N}=\left\{\begin{array}{c} \inf\left\{0\leq {t<\infty :∥\nabla U∥}_{L^{\infty }\left(R^{3}\right)}\geq N\right\},\\ \infty ,\;\mathrm{if}\;\mathrm{the}\;\mathrm{above}\;\mathrm{set}\;\{\cdots \}\;\mathrm{is}\;\mathrm{empty}. \end{array}\right. \end{array}$$

Then, we have $\tau_{N}={\tilde{\tau }}_{N},N=1,2,\cdots$ and $U(t\wedge \tau_{N})$ satisfies

(81) $$\begin{array}{c} U\left(t\wedge \tau_{N}\right)=U_{0}-\int_{0}^{t}\Pi B\left(U\right)I_{\left[0,\tau_{N}\right]}\left(s\right)ds+\Pi \sum_{j=1}^{\infty }\int_{0}^{t}G_{j}\left(s\right)I_{\left[0,\tau_{N}\right]}\left(s\right)dW_{j}\left(s\right), \end{array}$$

for all 0≤t<∞ and each $\omega \in \Omega_{0}$.

Thus, (U,τ) is a local smooth solution in the sense of Definition 1. We can show the uniqueness easily as in step 1. 

**Step 4.** Estimate of the stopping time. 

We now establish the estimate (11) of the stopping time. For the solution (U,τ) obtained in Step 3, fix any 0<δ<1, we have

$$\begin{array}{ccc} P\left\{\tau >\delta \right\}\geq P\left\{\tau_{N}>\delta \right\} & \geq & P\left\{\underset{0\leq t\leq \delta }{\sup}{∥\nabla U\left(t\wedge \tau_{N}\right)∥}_{L^{\infty }\left(R^{3}\right)}<N\right\}\\ & \geq & P\left\{\underset{0\leq t\leq \delta }{\sup}{∥U\left(t\wedge \tau_{N}\right)∥}_{\alpha }<KN\right\}, \end{array}$$

where K>0 ia a constant defined by

$$\begin{array}{c} K=\sup\left\{C\in {R:∥\nabla U\left(t\right)∥}_{L^{\infty }\left(R^{3}\right)}\leq \frac{1}{C}{∥U\left(t\right)∥}_{\alpha },\forall \;U\in H_{\sigma }^{\alpha }\right\}. \end{array}$$

Since $U\left(\cdot \wedge \tau_{N}\right)\in C\left(\left[0,\infty \right);H_{\sigma }^{\alpha }\right)$ for almost all ω, we can define a stopping time

$$\begin{array}{c} \tau_{L}=\left\{\begin{array}{c} \inf\left\{0\leq t<\infty :∥U\left(t\wedge \tau_{N}\right){∥}_{\alpha }\geq L\right\},\\ \tau_{N},\;\mathrm{if}\;\mathrm{the}\;\mathrm{above}\;\mathrm{set}\;\left\{\cdots \right\}\;\mathrm{is}\;\mathrm{empty}. \end{array}\right. \end{array}$$

Applying (14)–(16) with α+1 replaced by α and the Itô formula, we have

(82) $$\begin{array}{ccc} ∥U\left(t\wedge \tau_{L}\right){∥}_{\alpha }^{2} & \leq & ∥U_{0}{∥}_{\alpha }^{2}+CN\int_{0}^{t\wedge \tau_{L}}{∥U\left(s\right)∥}_{\alpha }^{2}ds\\ \; & + & 2\sum_{j=1}^{\infty }\int_{0}^{t\wedge \tau_{L}}{\langle U\left(s\right),G_{j}\left(s\right)\rangle }_{\alpha }dW_{j}\left(s\right)+\sum_{j=1}^{\infty }\int_{0}^{t\wedge \tau_{L}}{∥G_{j}\left(s\right)∥}_{\alpha }^{2}ds, \end{array}$$

for all 0≤t<∞, where C>0 is a constant independent of U,L,N,t. By the Burkholder–Davis–Gundy inequality,

(83) $$\begin{array}{ccc} \; & \; & E\left(\underset{0\leq \xi \leq t}{\sup}|\sum_{j=1}^{\infty }\int_{0}^{\xi \wedge \tau_{L}}{\langle U\left(s\right),G_{j}\left(s\right)\rangle }_{\alpha }dW_{j}\left(s\right)|\right)\\ \; & \leq & CE{\left(\sum_{j=1}^{\infty }\int_{0}^{t\wedge \tau_{L}}{∥U\left(s\right)∥}_{\alpha }^{2}{∥G_{j}\left(s\right)∥}_{\alpha }^{2}ds\right)}^{1/2}\\ \; & \leq & \frac{1}{2}E(\underset{0\leq s\leq t}{\sup}∥U\left(s\wedge \tau_{L}\right){∥}_{\alpha }^{2})+CE\left(\sum_{j=1}^{\infty }\int_{0}^{t\wedge \tau_{L}}{∥G_{j}\left(s\right)∥}_{\alpha }^{2}ds\right), \end{array}$$

for all 0≤t<∞, where C>0 is a constant independent of U,L,N,t. By (82), (83) and the Grönwall inequality, we have

$$\begin{array}{c} E\left(\underset{0\leq t\leq \delta }{\sup}{∥U\left(s\wedge \tau_{L}\right)∥}_{\alpha }^{2}\right)\leq Ce^{CN\delta }\left(E∥U_{0}{∥}_{\alpha }^{2}+\sum_{j=1}^{\infty }\int_{0}^{\delta }E{∥G_{j}\left(s\right)∥}_{\alpha }^{2}ds\right). \end{array}$$

As $\tau_{N}=\lim_{L\to \infty }\tau_{L}$, then we have

$$\begin{array}{c} E\left(\underset{0\leq t\leq \delta }{\sup}{∥U\left(s\wedge \tau_{N}\right)∥}_{\alpha }^{2}\right)\leq Ce^{CN\delta }\left(E∥U_{0}{∥}_{\alpha }^{2}+\sum_{j=1}^{\infty }\int_{0}^{\delta }E{∥G_{j}\left(s\right)∥}_{\alpha }^{2}ds\right) \end{array}$$

by passing L→∞. Choosing an integer *N* such that $\frac{1}{N}\leq \delta \leq \frac{1}{N-1}$, we have

$$\begin{array}{c} P\left\{\underset{0<t<\delta }{\sup}{∥U\left(t\wedge \tau_{N}\right)∥}_{\alpha }<KN\right\}\\ \geq 1-\frac{1}{K^{2}N^{2}}Ce^{CN\delta }\left(E∥U_{0}{∥}_{\alpha }^{2}+\sum_{j=1}^{\infty }\int_{0}^{\delta }E{∥G_{j}\left(s\right)∥}_{\alpha }^{2}ds\right)\\ \geq 1-C\delta^{2}\left(E∥U_{0}{∥}_{\alpha }^{2}+\sum_{j=1}^{\infty }\int_{0}^{\delta }E{∥G_{j}\left(s\right)∥}_{\alpha }^{2}ds\right), \end{array}$$

where *C* is a positive constant independent of U,δ. The proof of Theorem 1 is complete.
